# Metabarcoding reveals unique microbial mat communities and evidence of biogeographic influence in low‐oxygen, high‐sulfur sinkholes and springs

**DOI:** 10.1002/ece3.11162

**Published:** 2024-03-24

**Authors:** Davis Fray, Callahan A. McGovern, Dale A. Casamatta, Bopaiah A. Biddanda, Sarah E. Hamsher

**Affiliations:** ^1^ Annis Water Resources Institute Grand Valley State University Muskegon Michigan USA; ^2^ Department of Biology University of North Florida Jacksonville Florida USA; ^3^ Department of Biology Grand Valley State University Allendale Michigan USA

**Keywords:** 16S, biofilm, biogeography, cyanobacteria, diatoms, *rbc*L

## Abstract

High‐sulfur, low‐oxygen environments formed by underwater sinkholes and springs create unique habitats populated by microbial mat communities. To explore the diversity and biogeography of these mats, samples were collected from three sites in Alpena, Michigan, one site in Monroe, Michigan, and one site in Palm Coast, Florida. Our study investigated previously undescribed eukaryotic diversity in these habitats and further explored their bacterial communities. Mat samples and water parameters were collected from sulfur spring sites during the spring, summer, and fall of 2022. Cyanobacteria and diatoms were cultured from mat subsamples to create a culture‐based DNA reference library. Remaining mat samples were used for metabarcoding of the 16S and *rbc*L regions to explore bacterial and diatom diversity, respectively. Analyses of water chemistry, alpha diversity, and beta diversity articulated a range of high‐sulfur, low‐oxygen habitats, each with distinct microbial communities. Conductivity, pH, dissolved oxygen, temperature, sulfate, and chloride had significant influences on community composition but did not describe the differences between communities well. Chloride concentration had the strongest correlation with microbial community structure. Mantel tests revealed that biogeography contributed to differences between communities as well. Our results provide novel information on microbial mat composition and present evidence that both local conditions and biogeography influence these unique communities.

## Graphical abstract

Groundwater‐fed underwater sinkholes and springs create habitats with high‐sulfur, low‐oxygen conditions. Multi‐marker metabarcoding was used to describe microbial mat communities in these extreme aquatic habitats. Unique bacterial and diatom communities were found in five springs in Michigan and Florida, with water parameters and biogeography contributing to these differences.

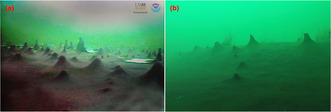

## 1 INTRODUCTION

Areas of karst geology are found throughout the Laurentian Great Lakes (Biddanda et al., 2006) and Florida (Barrios, 2006). In these regions, high‐sulfur, low‐oxygen groundwater dissolves surrounding bedrock and is released at the surface through springs and sinkholes (Biddanda et al., 2006). These conditions produce harsh environments that are often dominated by microbial mats (Franks & Stolz, 2009; Voorhies et al., 2012), which are thin, horizontally stratified layers of microbes over the sediment (Stal, 1995). While microbial mats can be found in a variety of habitats today, they are often the only life form able to tolerate the conditions in extreme aquatic environments (Prieto‐Barajas et al., 2018). Microbial mats are of interest because they are analogous to the communities that lived in ancient seas and contributed to the oxygenation of Earth's atmosphere (Dick et al., 2018). Such sites include sulfur springs, hot springs, and Antarctic lakes, providing similar habitats to those where Earth's most ancient life was found (Allwood, 2016). Modern microbial mats employ a variety of metabolic strategies to ensure their survival under these unique conditions (Biddanda et al., 2023; Canfield & Des Marais, 1993).

Cyanobacteria and sulfur‐oxidizing bacteria are dominant components of these microbial mat communities (Biddanda et al., 2012; Franks & Stolz, 2009; Stal, 1995). Diatoms, another primary producer, are the most common eukaryotes in some mat communities (Gomez et al., 2018; Perillo et al., 2022; Pinckney et al., 1995). In Middle Island Sinkhole (MIS), a submerged sulfur spring sinkhole in Michigan, motile taxa from microbial mats contribute to a complex, three‐dimensional mat structure featuring diurnal shifts in position to utilize a variety of metabolic strategies and exploit changing resources, truly a syntrophic system (Biddanda et al., 2015, 2023). In this habitat, cyanobacterial filaments dominate the top of the mat community during the day to exploit sunlight for photosynthesis, and *Craticula cuspidata*, a motile diatom, migrates vertically through the mats to store nitrogen in the absence of light for nitrogen respiration, giving them an advantage over non‐motile organisms in this environment (Biddanda et al., 2023; Merz et al., 2021). Archaea are found in microbial mat communities as well, particularly in the underlying sediment where their primary role may be methanogenesis (Nold, Pangborn, et al., 2010).

The isolated, unique conditions found in these types of spring habitats, along with their usually depauperate flora, present ideal circumstances for investigating microbial biogeography (Power et al., 2018). Biogeography, once expected to have minimal influence on microbial community structure (e.g., Bass Becking, 1934; Finlay & Fenchel, 2004), has been used recently to explain differences in floras that occur in disparate locations, especially aquatic environments (e.g., Dvořák et al., 2021; Filker et al., 2016; Kociolek et al., 2017; Ribeiro et al., 2018). In addition, describing biogeographic trends can contribute to an increased understanding of microbial dispersal, community structure, and composition (Burgsdorf et al., 2014; Lear et al., 2013).

DNA metabarcoding methods have proven useful to investigate diversity of microbial communities and have been used for exploring biogeographic trends (Antich et al., 2022; Pitz et al., 2020; Šupraha et al., 2022). Advantages of metabarcoding over the traditional light microscopy methods for identifying and quantifying algal communities include cost, reproducibility (Kermarrec et al., 2013), detection of species that may be overlooked in morphological assessments due to their small size or rarity (Pérez‐Burillo et al., 2022), clear identification of some groups of microbes (especially when reproductive structures are lacking), and an increasing lack of taxonomists available for this work (Kahlert et al., 2012). Metabarcoding studies have found higher diversity than detected with morphological methods in studies of cyanobacteria (e.g., Li et al., 2019) and diatoms (e.g., Zimmermann et al., 2015). Using a multi‐marker approach has revealed increased diversity across a wide range of taxa in bacterial and algal communities (Marcelino & Verbruggen, 2016; Wolf & Vis, 2019). The large subunit of the RUBISCO (*rbc*L) marker region has the ability to distinguish between diatom species (e.g., Apothéloz‐Perret‐Gentil et al., 2021; Hamsher et al., 2011), and the universal 16S V4 marker region has proven useful to detect cyanobacteria, other bacterial taxa, and Archaea (Walters et al., 2015).

In contrast to these advantages of metabarcoding, molecular surveys of microbial diversity remain limited by the lack of available reference sequences (Esenkulova et al., 2020; von Wintzingerode et al., 1997) and misidentification of taxa in reference databases (Dvořák et al., 2018; McGovern et al., 2023), issues that must be improved upon by pairing microbial culturing/sequencing efforts with taxonomy to overcome this barrier and make metabarcoding a viable strategy for ecological studies, especially long‐term monitoring efforts.

Previous research on microbial mats in these sulfur springs includes molecular surveys of diversity, such as analysis of small subunit ribosomal RNA clone libraries from the MIS and Great Sulfur Spring (GSS) in Michigan (Nold, Pangborn, et al., 2010; Nold, Zajack, et al., 2010; Chaudhary et al., 2009, respectively), pyrosequencing at MIS (Voorhies et al., 2012), and high‐throughput 16S rRNA sequencing at MIS (Grim et al., 2023; Kinsman‐Costello et al., 2017). Transcriptomics and proteomics have also been used to assess community composition and processes that community members are undergoing in MIS (Grim et al., 2021, 2023). Additionally, groundwater analyses have characterized the aquifer sources of MIS (Grim et al., 2023) and GSS (Haack et al., 2005). These studies have revealed the biogeochemical and metabolic processes occurring in these habitats, particularly in MIS, but a deeper look into the taxonomic composition of these sites and exploration of new spring habitats is merited to better describe these communities and factors that influence them.

For this study, multi‐marker metabarcoding analyses were performed targeting bacterial, archaeal, and diatom diversity to investigate the microbial mat communities of five low‐oxygen, high‐sulfur springs in Michigan and Florida. The main goals of this study were to (1) compare water parameters and microbial mat community diversity between these sites with unique conditions; (2) document undescribed taxonomic composition of microbial mat communities from sulfur spring sites using metabarcoding data supplemented by a culture‐based DNA reference library; and (3) explore whether environmental characteristics and/or geographic distance between springs drive any differences observed between these microbial mat communities.

## 2 METHODS

### 2.1 Sites

Three sites were investigated that lie in a region near Alpena, Michigan, wherein karst geology has led to the formation of numerous sinkholes and springs (Biddanda et al., 2006). MIS (45°11′54.2″ N 83°19′30.2″ W) is a 23‐m‐deep sinkhole in Lake Huron where cool (~10°C), high‐sulfur (>1000 mg/L), low‐oxygen (<1 mg/L) groundwater vents and pools in a basin, creating an isolated environment with unique conditions relative to surrounding waters (Biddanda et al., 2006, 2012). Similar environments are created in a nearshore shallow spring outlet in El Cajon Bay (ECB, 45°05′07.5″ N 83°19′28.3″ W) and an artesian well fountain in downtown Alpena (FTN, 45°03′44.9″ N 83°25′52.6″ W). Groundwater with nearly identical water parameters characterizes these sites and is thought to originate from a shared source (Snider et al., 2017). Differing levels of sunlight and surface water mixing occur at each site. In MIS, microbial mats receive only 5%–10% of the sunlight measured at the lake surface, while shallower mats at ECB (∼0.25–2 m) receive 50%–90% of this light (Biddanda et al., 2015). Mats from two spring habitats in ECB were sampled, a shallow spring at 0.25–0.5 m in depth, and a deeper spring at ~1 m. Each of the three tiers of FTN was sampled, and mat samples were collected from one area of MIS near its source, at a depth of 23 m.

A large sulfur spring sinkhole with a similar carbonate aquifer groundwater source to the Alpena sites, GSS (41°46′04.3″ N 83°27′21.7″ W), is surrounded by marshland along the shore of Lake Erie's western basin. The aquifer below dissolved the rock layers around it, forming a 13‐m‐deep sinkhole, wherein groundwater pours into a 42‐m‐wide, tufa‐rimmed pond (Chaudhary et al., 2009; Lundstrom et al., 2004). Spring water flows out of the pond through a culvert, a channel, and eventually emptying into Lake Erie. Mats were collected within the pond near the shoreline, near the spring source at 13 m deep, and at the outlet culvert.

Another accessible sulfur spring was in Washington Oaks Gardens State Park, Florida (OAK, 29°37′54.2″ N 81°12′30.3″ W). Groundwater flows out of an artesian well into a 4‐m‐wide bay, where it is contained by a ring of rocks and concrete. Floating microbial mats, benthic mats, and white filaments near the spring outlet were collected at this site.

### 2.2 Sample collection

Each site was visited in the spring (April–May), summer (June–July), and fall (September) periods. Exceptions include MIS and OAK, which were only sampled during the summer period. During each visit, a YSI multiprobe (Yellow Springs Instruments, Inc., Yellow Springs, OH, USA) was used to measure temperature (Temp), specific conductance (Cond.), and percent dissolved oxygen (ODO.). Due to multiprobe malfunction, data from a summer 2021 YSI deployment were used to characterize MIS water parameters. In addition to YSI parameters, 250 mL acid‐washed Nalgene bottles were used to collect water samples for nutrient analyses at each sampling point. Each water sample was subsampled into two vials, of which one was refrigerated and one was frozen within 24 h of collection. The refrigerated subsample was used to determine orthophosphate (SRP) concentrations using USEPA method 365.1 (O'Dell, 1996). The frozen subsample was used to determine dissolved silica concentrations using USEPA method 370.1 (USEPA, 1978) and chloride (Cl.mg.L), sulfate (SO_4_.mg.L), and nitrate using USEPA method 300.0 (Pfaff, 1993).

Mats from wadable sites were collected using a suction device and placed in sterile Whirlpak® bags, and then put on ice for transport to the Annis Water Resources Institute (AWRI, Muskegon, MI, USA). Three replicate mat samples were collected from each habitat type at each site during each sampling event. Mats from MIS were collected by NOAA divers using a coring device and transported to AWRI as intact cores in plastic tubes on ice. Mats from the source of GSS (13 m) were visualized using an Eyoyo underwater camera (Eyoyo Ltd, Shenzen, China) and collected during the fall sampling period using a 15 m aluminum pole with a 20 μm plankton net affixed to the end for gathering intact mats, with the aid of the underwater camera to guide sampling efforts and ensure representative mat sample collection. Plankton tow samples were also collected at GSS and ECB to determine taxa that may be considered part of the surrounding planktonic community, rather than active members of the microbial mat community. Each mat sample collected was subsampled, with one subsample used for generating unialgal cultures and the other for metabarcoding.

### 2.3 Culture‐based DNA reference library

Similar to the strategy employed in Hamsher et al. (2013), individual diatom cells were isolated from each culturing subsample via micropipette serial dilution to establish unialgal cultures. Monocultures were maintained in WC + Si liquid medium (Guillard & Lorenzen, 1972) at 10°C and a 12:12 light cycle. For morphological identification of cultures, live material was boiled in HNO_3_ for 1 h, repeatedly washed and settled with ddH_2_O, dried on coverslips, and mounted on slides using Naphrax®. Each culture was identified to species under 1000× using a Nikon Eclipse Ni‐U light microscope with DIC and Krammer and Lange‐Bertalot (1986, 1988, 1991a, 1991b). When monocultures had grown to a sufficient density for DNA extraction, cells were harvested by centrifugation and a Chelex extraction was performed following Richlen and Barber (2005). The *rbc*L region of each culture was amplified using primers *rbc*L66+ (Alverson et al., 2007) and DP*rbc*L7‐ (Jones et al., 2005), Cytiva PuReTaq™ Ready‐To‐Go™ PCR beads (Cytiva, Marlborough, MA, USA), and a thermocycler protocol of 94°C for 3 min 30 s, then 36 cycles of 94°C for 50 s, 52°C for 50 s, 72°C for 80 s, with a final extension at 72°C for 15 min (Stepanek et al., 2015). The PCR products were frozen and sent to Eurofins Scientific (Louisville, Kentucky) for Sanger sequencing using the PCR primers as well as internal primers CfD+ (Hamsher et al., 2011) and *rbc*L1255‐ (Alverson et al., 2007). Sequences were assembled, edited, and aligned using Geneious Prime (Version 11.0.15+10). The final alignment of *rbc*L sequences included data from 43 cultures (~1370 bp with no indels).

To isolate cyanobacterial taxa, mat samples were spread onto solid Z‐8 medium (Rippka, 1988) and nitrogen‐free Z‐8 medium to isolate a wider range of cyanobacteria, and grown under ambient conditions (23°C, ∼16:8 h light: dark photoperiod). Colonies were individually picked and plated until unialgal cultures were achieved. Morphology of the strains was analyzed via light microscopy (Nikon Eclipse N*i* with DIC), and taxonomic identification was assessed using Wehr et al. (2015) and Komárek and Anagnostidis (2005). Images were taken with a high‐resolution camera (Nikon digital sight DS‐U3). Direct PCR was performed as follows: cells were placed at −20°C for 30 min, centrifuged, and the supernatant containing DNA collected. The partial 16S rRNA gene (hereafter abbreviated as 16S) and the whole 16S‐23S ITS region (Gaylarde et al., 2004) were amplified using primers CYA8F and CYAB23R (Neilan et al., 1997). The 50 μL PCR reaction contained: 27 μL DNA containing supernatant, 0.5 μL of each primer (0.01 mM concentration), and 22 μL PCR Master Mix (Promega, Madison, WI, USA). PCR amplification proceeded as detailed in Casamatta et al. (2005), and products were frozen and sent to Eurofins Scientific (Louisville, Kentucky) for Sanger sequencing.

### 2.4 Metabarcoding

Subsamples for metabarcoding were frozen at −80°C within 36 h of collection, except for MIS samples which were stored at 10°C for 72 h prior to harvesting, then frozen at −80°C, due to logistical limitations. DNA was extracted from the metabarcoding subsamples using the Qiagen PowerSoil DNA Extraction Kit (Qiagen, Crawley, UK) according to the manufacturer's protocol, with a negative control consisting of autoclaved nanopore water included for each subset of extractions and for each primer to assess potential processing contamination. To prepare samples for Illumina amplicon sequencing, a two‐step PCR approach was employed. The initial PCR was completed to amplify the two barcode markers (*rbc*L and 16S) in individual reactions using specific primers with the attached Illumina adapter. The primary PCR amplification was completed in 25 μL reactions using 12.5 μL of Q5 High‐Fidelity2X Master Mix (New England BioLabs Inc., Ipswich, MA, USA), 1.0 μL of each primer (1 μM), 9.5 μL RNase‐free H2O, and 1 μL DNA. For the 16S marker, the primer pair and thermocycler protocol from Walters et al. (2015) were employed. For the *rbc*L marker, we targeted a 312 bp region of the *rbc*L plastid gene using an equimolar mix of the three forward and two reverse degenerate primers from Vasselon et al. (2017), along with their thermocycler protocol.

Following PCR amplification, samples were sent to the University of Tennessee, Knoxville, for processing and sequencing. PCR products were cleaned with Agencourt AmPure XP beads (Beckman Coulter Inc., Indianapolis, IN, USA) and quantified using a Qubit Fluorometer (v.2.0; ThermoFisher Scientific, Waltham, MA, USA). Samples were normalized, and a second PCR reaction (50 μL) enriched with Q5 High‐Fidelity 2X Master Mix was performed to apply indexing primers, following cycling conditions: 95°C for 3 min followed by 10 cycles of 95°C for 30 s, 55°C for 30 s, 72°C for 30 s, with a final extension of 72°C for 5 min, modified from the 16S protocol (Illumina, 2013). A second PCR clean‐up was performed, and samples were quantified using a Qubit Fluorometer. Libraries were loaded with 25% PhiX clustering control on the Illumina MiSeq platform for 300 bp *×* 2 paired‐end reads using the V3 kit.

The resulting sequence datasets were analyzed separately for each marker region. Sequences were demultiplexed and adapters were removed. Primers were trimmed using Cutadapt version 4.2 (Martin, 2011). Using the DADA2 pipeline (Callahan et al., 2016), reads were quality filtered based on Q30 scores and trimmed to remove low‐quality reads. Filtered reads were denoised and dereplicated using DADA2 to produce amplicon sequence variants (ASVs). Singletons, doubletons, and chimeric sequences were removed from the dataset. ASVs identified as chloroplast or mitochondria in the 16S dataset were removed. The SILVA database (release 138.1, Quast et al., 2013) appended with CyanoSeq (Lefler et al., 2023) was employed to assign taxonomy to the 16S ASVs. For the *rbc*L dataset, taxonomy was assigned using the curated reference database Diat.barcode (Rimet et al., 2019). For both datasets, ASVs matching our culture‐generated sequences were assigned to the taxa we identified them as, and reference taxonomy assignment (from SILVA/CyanoSeq or Diat.barcode) was replaced if taxonomy assignment differed. Only ASVs assigned to diatom taxa were kept for the *rbc*L marker. Two genera found to dominate the plankton tow samples, *Cyclotella* and *Lindavia*, were removed from the *rbc*L data analyses because they are planktonic taxa and unlikely to be active members of the mat community.

### 2.5 Statistics

RStudio (v4.4.4; R Core Team, 2022) was used for statistical analyses of the resulting water parameters and metabarcoding data. Water parameters were Tukey‐transformed prior to statistical comparisons. Measures that fell below the detection limit were included as zeros in statistical analyses. All variables were tested for normality and homoscedasticity using Shapiro tests and Bartlett tests, respectively, with the vegan package (v2.6.4; Oksanen et al., 2022). To compare water parameters between sites, Welch analysis of variance (ANOVAs) and Games‐Howell post‐hoc comparisons were run using the vegan package (v2.6.4; Oksanen et al., 2022). Kruskal‐Wallis rank‐sum tests were used for water parameters with non‐normal distributions (conductivity, dissolved oxygen, nitrate) using the agricolae package (v1.3.5; de Mendiburu, 2021), and post‐hoc Dunn tests were run using the FSA package (v0.9.4; Ogle et al., 2023). Statistical analyses of diversity were performed separately for each molecular marker (16S and *rbc*L). Observed (ASV richness) and Shannon alpha diversity metrics were calculated for each site using the phyloseq package (v1.42.0; McMurdie & Holmes, 2013). To compare alpha diversity between sites, Kruskal–Wallis rank‐sum tests were used for measures with non‐normal distributions (16S Observed, *rbc*L Observed, *rbc*L Shannon) using the agricolae package (v1.3.5; de Mendiburu, 2021), and post‐hoc Dunn tests were run using the FSA package (v0.9.4; Ogle et al., 2023). The 16S Shannon diversity values were compared using one‐way analysis of variance (ANOVA) and Tukey's post‐hoc comparison using the vegan package (v2.6.4; Oksanen et al., 2022).

Microbial community composition was compared by generating Bray–Curtis community dissimilarity matrices for each sample and running a permutational analysis of variance (PERMANOVA) test to investigate differences between sites using the microViz package (v0.10.7; Barnett et al., 2021). A post‐hoc pairwise PERMANOVA was run to determine whether sites differed from one another using the pairwiseAdonis package (v0.4.1; Martinez Arbizu, 2020). To investigate the influence of environmental parameters on community composition, significance of variables was tested using the function envfit of the vegan package (v2.6.4; Oksanen et al., 2022), which through multiple regression indicated that all variables were significantly related to the ordination axes (*p* < .05). An RDA ordination for each marker was plotted with these variables along with taxa that contributed most to the axes using the microViz package (v0.10.7; Barnett et al., 2021). Spearman's rank correlation coefficients were calculated to assess correlations between water parameters and taxa using the ggpubr package (v0.6.0; Kassambara, 2023). To further explore environmental influences and compare them to the effects of geographic distance between sites, Mantel tests were performed on the environmental data, geographic distances generated using the geosphere package (v1.5.18; Hijmans, 2022), and Bray–Curtis community dissimilarity matrices to determine the significance and relative influence of these variables with the vegan package (v2.6.4; Oksanen et al., 2022). To visualize taxonomic composition of OAK and GSS, heatmaps were generated using Hellinger‐transformed relative abundances of taxa with >5% prevalence using the microViz package (v0.10.7; Barnett et al., 2021).

## 3 RESULTS

### 3.1 Water parameters

Water parameters measured varied statistically between sites (Table 1), except for percent dissolved oxygen (*H*_4_ = 8.79, *p =* .067). Temperature ranged from 8.90 to 15.18°C in Michigan sites and was significantly warmer at OAK (23.0–23.4°C, *F*_4,16_ = 24.7, *p* < .001). Conductivity was lowest at MIS, intermediate at ECB, FTN, and GSS, and highest at OAK (*H*_4_ = 15.2, *p* = .004). MIS had significantly higher pH (*F*_4,16_ = 5.9, *p* = .004) than the other sites. Sulfate showed a gradient of differing concentrations (*F*_4,16_ = 4.3, *p* = .015) ranging from lowest at OAK, intermediate at ECB, MIS, and FTN, and highest at GSS. A gradient of chloride concentrations (*F*_4,16_ = 46.2, *p* < .001) was found, from lowest at MIS, to intermediate at ECB, GSS, and FTN, to highest at OAK. Dissolved silica also showed a gradient of concentrations, from highest at OAK, to intermediate at FTN and GSS, to lowest at ECB (*F*_3,15_ = 17.9, *p* < .001). Nitrate concentrations were higher at OAK and ECB than at the other sites (*H*_4_ = 12.3, *p =* .020), with FTN, GSS, and MIS samples never exceeding the detection limit (0.01 mg/L). No soluble reactive phosphorus (SRP) concentrations were found above the detection limit (0.005 mg/L).

**TABLE 1:** Water parameters measured for each site, reported as mean (range).

Parameter	Sites				
	MIS (*n* = 2)	ECB (*n* = 6)	FTN (*n* = 3)	GSS (*n* = 8)	OAK (*n* = 2)
Temp. (°C)	9.25^C^ (8.95–9.54)	9.95^C^ (8.90–10.58)	11.71^BC^ (11.23–12.30)	13.38^B^ (11.42–15.18)	23.20^A^ (23.0–23.4)
Specific conductance (μS/cm)	2038.5^B^ (2015–2062)	2284.5^AB^ (1604–2573)	2497.7^AB^ (2446–2554)	2588.3^AB^ (2504–2629)	5255.0^A^ (4670–5840)
pH	7.99^A^ (7.99–8.00)	7.26^BC^ (7.04–7.85)	7.42^B^ (7.40–7.44)	7.35^B^ (7.12–7.74)	7.00^C^
Dissolved oxygen (%)	11.6^A^ (11.4–11.8)	12.0^A^ (6.2–27.7)	29.9^A^ (25.4–32.5)	37.8^A^ (6.2–57.5)	7.5^A^ (7.2–7.8)
Sulfate (mg/L)	942.0^AB^ (716–1168)	952.2^AB^ (656–1206)	1089.3^AB^ (1011–1134)	1180.1^A^ (1059–1327)	632.5^B^ (626–639)
Chloride (mg/L)	20.5^D^ (18–23)	48.2^BC^ (32–63)	77.0^B^ (76–79)	37.6^C^ (33–44)	1842.0^A^
Silica (mg/L)	–	9.61^B^ (8.42–10.84)	11.43^AB^ (10.36–13.34)	11.33^AB^ (10.27–12.27)	16.01^A^ (15.14–16.88)
Nitrate (mg/L)	<0.01^B^	0.016^AB^ (<0.01–0.51)	<0.01^B^	<0.01^B^	0.755^A^ (0.36–1.15)
SRP (mg/L)	<0.005	<0.005	<0.005	<0.005	<0.005

*Note*: Values sharing a superscript letter are not significantly different for that parameter. Most nitrate and all soluble reactive phosphorus (SRP) concentrations fell below the detection limits of 0.01 and 0.005 mg/L, respectively. Silica concentration was not measured for MIS.

Abbreviations: ECB, El Cajon Bay; FTN, Alpena Fountain; GSS, Great Sulfur Spring; MIS, Middle Island Sinkhole; OAK, Florida Oak Spring.

### 3.2 Microbial mats

**FIGURE 1:**
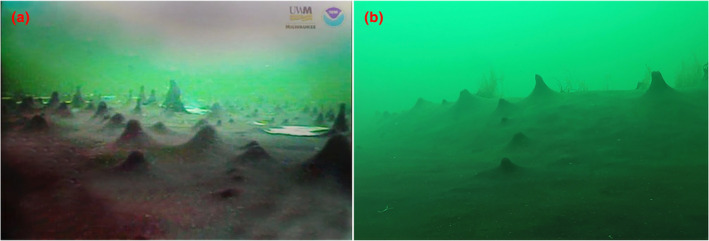
Comparison of underwater imagery of microbial mats found in: (a) = Middle Island Sinkhole, Alpena, MI (Rob Paddock, University of Wisconsin), (b) = Great Sulfur Spring, Erie, MI.

Microbial mat growth was found at all sites but was limited during the spring collection period at ECB. At GSS, underwater photography was used to observe microbial mat growth near its source at 13 m depth. The camera revealed lawn‐like, purple microbial mat growth in the area surrounding the outlet at GSS, with finger‐like structures created by gases underneath the mat, a macroscopically similar community to those documented at MIS (Figure 1; Biddanda et al., 2015). Some mats found at ECB and FTN were also purple, but the FTN mats were notably thicker and included more white filamentous growth. Mats at OAK appeared largely composed of filamentous white bacteria, with floating mats showing a mixture of purple, gray, and green coloration macroscopically.

### 3.3 Metabarcoding

The 16S marker yielded 4,271,473 paired‐end reads (*n* = 70 samples), while the *rbc*L marker produced 3,185,738 paired‐end reads (*n* = 86 samples). Reads were assigned to 23,427 unique 16S amplicon sequence variants (ASVs) and 2043 *rbc*L ASVs. Taxonomy assignment for the 16S marker resulted in 16,612 ASVs (70.1%) identified as family or lower taxonomic level. Taxonomy assignment for the *rbc*L marker resulted in 1338 ASVs (65.5%) with a genus‐ or species‐level identification. Sequences matching cultured diatoms accounted for 34.4% of the *rbc*L reads. Bacterial and diatom genera sequenced from each site are provided in Tables 2 and 3, respectively.

**TABLE 2:** Bacteria genera/identifiers present (X) or absent (−) in each site.

Order	Genus/Identifier	FTN	ECB	GSS	MIS	OAK
Abditibacteriales	*Abditibacterium*	–	X	X	X	X
Acanthopleuribacterales	*Acanthopleuribacter*	–	–	–	–	X
Acetobacterales	*Acidocella*	–	X	–	–	–
Acetobacterales	*Rhodovastum*	–	X	X	X	–
Acetobacterales	*Roseococcus*	–	–	–	X	X
Acetobacterales	*Roseomonas*	X	X	X	X	X
Acholeplasmatales	EUB33‐2	–	–	–	X	–
Acidiferrobacterales	*Sulfurifustis*	–	X	X	X	–
Acidobacteriales	*Acidipila‐Silvibacterium*	–	–	–	–	X
Acidobacteriales	*Terriglobus*	–	–	–	–	X
Alicyclobacillales	*Alicyclobacillus*	–	X	–	–	–
Alicyclobacillales	*Tumebacillus*	–	X	X	–	–
Alphaproteobacteria Incertae Sedis	*Acuticoccus*	–	–	–	–	X
Altiarchaeales	*Candidatus* Altiarchaeum	X	X	X	X	X
Anaerolineales	*Anaerolinea*	X	X	X	X	X
Anaerolineales	Anaerolineaceae UCG‐001	–	X	X	–	X
Anaerolineales	GWD2‐49‐16	–	X	X	X	–
Anaerolineales	*Leptolinea*	X	X	X	X	X
Anaerolineales	*Levilinea*	–	–	X	X	X
Anaerolineales	*Longilinea*	–	X	X	X	X
Anaerolineales	*Ornatilinea*	–	–	X	X	–
Anaerolineales	*Pelolinea*	–	X	X	–	X
Anaerolineales	RBG‐16‐58‐14	–	X	X	–	–
Anaerolineales	*Thermomarinilinea*	–	–	–	–	X
Anaerolineales	UTCFX1	–	X	X	–	–
Arenicellales	*Candidatus* Thiosymbion	–	–	–	–	X
Arenicellales	HTCC5015	–	X	–	–	–
Azospirillales	*Skermanella*	–	–	X	–	X
Bacillales	*Bacillus*	–	X	X	X	X
Bacillales	*Domibacillus*	–	X	–	–	–
Bacillales	*Fictibacillus*	–	X	X	–	–
Bacillales	*Kurthia*	–	–	X	–	–
Bacillales	*Lysinibacillus*	–	–	X	–	–
Bacillales	*Paenisporosarcina*	–	X	X	–	–
Bacillales	*Planomicrobium*	–	–	X	–	–
Bacillales	*Psychrobacillus*	–	X	X	–	–
Bacillales	*Sporolactobacillus*	–	X	–	–	–
Bacillales	*Sporosarcina*	–	X	X	–	–
Bacteriovoracales	*Bacteriovorax*	X	X	X	X	–
Bacteriovoracales	*Peredibacter*	–	X	X	X	X
Bacteroidales	[*Cytophaga*] xylanolytica group	X	X	X	X	–
Bacteroidales	Acetobacteroides	–	X	X	X	X
Bacteroidales	Bacteroides	–	X	X	X	X
Bacteroidales	Blvii28 wastewater‐sludge group	X	X	X	X	X
Bacteroidales	BSV13	–	X	X	X	–
Bacteroidales	*Carboxylicivirga*	–	–	–	–	X
Bacteroidales	*Dysgonomonas*	–	X	X	–	X
Bacteroidales	GWE2‐42‐42	–	X	X	X	X
Bacteroidales	*Labilibacter*	–	–	–	–	X
Bacteroidales	Macellibacteroides	–	X	X	X	X
Bacteroidales	Mangrovibacterium	–	–	–	–	X
Bacteroidales	*Mangroviflexus*	–	X	–	X	–
Bacteroidales	*Meniscus*	–	X	–	–	–
Bacteroidales	*Mucinivorans*	–	X	X	–	–
Bacteroidales	*Paludibacter*	X	X	X	X	X
Bacteroidales	*Prevotella*	–	X	–	–	–
Bacteroidales	*Prevotella* 9	–	–	X	–	–
Bacteroidales	*Rikenella*	–	X	–	–	–
Bacteroidales	*Roseimarinus*	–	X	X	X	X
Bacteroidales	*Sunxiuqinia*	–	–	–	–	X
Bacteroidales	WCHB1‐32	X	X	X	X	–
Bacteroidales	*Williamwhitmania*	–	X	X	X	–
Balneolales	*Balneola*	–	–	–	–	X
Balneolales	*Gracilimonas*	–	X	X	–	–
Balneolales	*Soortia*	–	–	–	–	X
Bdellovibrionales	*Bdellovibrio*	X	X	X	X	X
Bdellovibrionales	OM27 clade	–	X	X	X	X
Beggiatoales	*Beggiatoa*	X	X	X	X	X
Beggiatoales	*Thioflexothrix*	–	–	–	–	X
Blastocatellales	*Aridibacter*	–	–	–	–	X
Blastocatellales	*Blastocatella*	X	–	–	X	X
Blastocatellales	JGI 0001001‐H03	–	X	X	X	–
Blastocatellales	*Stenotrophobacter*	–	–	X	X	–
Brevibacillales	*Brevibacillus*	–	X	X	–	–
Brevinematales	*Brevinema*	–	X	X	X	X
Bryobacterales	*Bryobacter*	X	X	X	X	X
Burkholderiales	966‐1	–	X	X	X	X
Burkholderiales	*Aquaspirillum*	–	–	X	–	–
Burkholderiales	*Aquincola*	–	–	–	X	–
Burkholderiales	*Burkholderia‐Caballeronia‐Paraburkholderia*	–	X	–	–	–
Burkholderiales	*Candidatus* Accumulibacter	X	X	X	X	–
Burkholderiales	*Candidatus* Nitrotoga	–	X	X	–	–
Burkholderiales	*Candidatus* Symbiobacter	–	–	X	–	–
Burkholderiales	*Chitinibacter*	–	–	X	–	–
Burkholderiales	*Chitinilyticum*	–	X	X	–	–
Burkholderiales	*Chitinimonas*	–	–	X	–	–
Burkholderiales	*Chitiniphilus*	–	–	X	–	–
Burkholderiales	*Chromobacterium*	–	–	–	–	X
Burkholderiales	*Collimonas*	–	–	X	–	–
Burkholderiales	*Crenobacter*	–	X	–	–	–
Burkholderiales	*Dechlorobacter*	–	–	–	–	X
Burkholderiales	*Dechloromonas*	–	X	X	X	–
Burkholderiales	*Deefgea*	–	X	X	X	–
Burkholderiales	*Denitratisoma*	–	X	–	–	–
Burkholderiales	DSSD61	–	X	X	X	–
Burkholderiales	Ellin6067	–	X	X	X	–
Burkholderiales	*Ferriphaselus*	–	X	–	–	–
Burkholderiales	*Ferritrophicum*	–	X	X	–	–
Burkholderiales	*Formivibrio*	–	X	X	–	X
Burkholderiales	*Gallionella*	–	X	–	–	–
Burkholderiales	*Georgfuchsia*	–	X	–	X	–
Burkholderiales	*Giesbergeria*	–	–	X	–	–
Burkholderiales	GOUTA6	–	X	X	X	–
Burkholderiales	*Herminiimonas*	–	–	X	–	–
Burkholderiales	*Hydrogenophaga*	X	X	X	X	X
Burkholderiales	*Hylemonella*	–	–	–	–	X
Burkholderiales	*Ideonella*	–	X	X	–	X
Burkholderiales	*Inhella*	–	X	X	–	–
Burkholderiales	*Iodobacter*	–	X	X	X	–
Burkholderiales	IS‐44	–	X	X	X	–
Burkholderiales	*Leeia*	–	X	–	–	–
Burkholderiales	*Leptothrix*	X	X	X	X	–
Burkholderiales	*Limnobacter*	–	X	X	–	–
Burkholderiales	*Limnohabitans*	–	–	–	–	–
Burkholderiales	*Massilia*	–	X	X	–	–
Burkholderiales	*Methylotenera*	X	X	X	X	–
Burkholderiales	*Methyloversatilis*	–	–	X	–	–
Burkholderiales	*Microvirgula*	–	–	X	–	–
Burkholderiales	mle1‐7	–	X	X	–	X
Burkholderiales	MM1	–	–	–	X	–
Burkholderiales	MND1	–	X	X	X	–
Burkholderiales	*Nitrosomonas*	–	–	X	–	–
Burkholderiales	*Nitrosospira*	–	–	–	–	–
Burkholderiales	*Niveibacterium*	–	X	X	–	–
Burkholderiales	*Noviherbaspirillum*	–	X	X	–	–
Burkholderiales	*Paludibacterium*	–	–	X	–	–
Burkholderiales	*Paucibacter*	–	X	X	–	–
Burkholderiales	*Piscinibacter*	–	X	X	–	–
Burkholderiales	*Polaromonas*	–	X	X	X	–
Burkholderiales	*Polynucleobacter*	–	X	–	–	X
Burkholderiales	*Procabacter*	–	–	X	–	–
Burkholderiales	*Propionivibrio*	X	X	–	–	X
Burkholderiales	*Rhizobacter*	X	X	X	X	–
Burkholderiales	*Rhodoferax*	X	X	X	X	–
Burkholderiales	*Rivibacter*	–	–	–	X	–
Burkholderiales	*Rubrivivax*	–	–	–	–	X
Burkholderiales	*Sideroxydans*	–	X	–	X	–
Burkholderiales	*Simplicispira*	–	–	X	–	–
Burkholderiales	*Sphaerotilus*	–	X	X	–	X
Burkholderiales	*Sulfuricella*	–	X	X	–	–
Burkholderiales	*Sulfuriferula*	X	–	–	–	–
Burkholderiales	*Sulfurisoma*	–	–	–	X	–
Burkholderiales	*Sulfuritalea*	–	X	–	X	–
Burkholderiales	*Thiobacillus*	X	X	X	X	X
Burkholderiales	*Uliginosibacterium*	–	X	X	–	X
Burkholderiales	*Undibacterium*	–	X	–	–	–
Burkholderiales	*Variovorax*	–	–	X	–	–
Burkholderiales	*Vogesella*	–	–	X	–	–
Caedibacterales	*Caedibacter*	–	–	X	–	–
Caldilineales	*Litorilinea*	–	–	X	–	–
Caldisericales	*Caldisericum*	–	–	X	X	–
Calditrichales	*Caldithrix*	X	X	X	–	X
Calditrichales	*Calorithrix*	–	X	X	–	–
Calditrichales	JdFR‐76	–	X	–	–	–
Calditrichales	SM23‐31	X	X	X	X	X
Campylobacterales	*Arcobacter*	–	–	X	–	–
Campylobacterales	*Pseudarcobacter*	–	–	X	–	–
Campylobacterales	*Sulfuricurvum*	X	X	–	X	–
Campylobacterales	*Sulfurimonas*	–	–	X	X	–
Campylobacterales	*Sulfurospirillum*	X	X	X	X	–
Campylobacterales	*Sulfurovum*	X	X	X	X	X
Caulobacterales	*Amphiplicatus*	–	X	X	–	–
Caulobacterales	*Asticcacaulis*	–	X	X	–	X
Caulobacterales	*Brevundimonas*	X	X	X	X	–
Caulobacterales	*Caulobacter*	X	X	X	X	X
Caulobacterales	*Hirschia*	X	X	X	X	X
Caulobacterales	*Hyphomonas*	X	X	X	X	X
Caulobacterales	*Marinicaulis*	–	–	–	–	X
Caulobacterales	*Parvularcula*	–	–	–	–	X
Caulobacterales	*Phenylobacterium*	–	–	X	X	–
Caulobacterales	*Ponticaulis*	–	–	–	–	X
Caulobacterales	SWB02	–	X	X	X	X
Caulobacterales	UKL13‐1	–	X	–	–	X
Chitinivibrionales	Possible genus 03	–	X	–	–	–
Chitinophagales	*Aurantisolimonas*	–	X	X	X	X
Chitinophagales	*Aureispira*	X	X	X	–	–
Chitinophagales	*Chitinophaga*	–	–	–	–	X
Chitinophagales	*Dinghuibacter*	–	X	X	X	X
Chitinophagales	*Edaphobaculum*	–	X	X	–	X
Chitinophagales	*Ferruginibacter*	X	X	X	X	–
Chitinophagales	*Flavihumibacter*	–	X	X	–	–
Chitinophagales	*Flavisolibacter*	–	–	X	–	–
Chitinophagales	*Haliscomenobacter*	X	X	X	X	X
Chitinophagales	*Lacibacter*	–	X	X	X	–
Chitinophagales	*Lewinella*	X	X	X	X	X
Chitinophagales	*Niastella*	–	–	X	–	–
Chitinophagales	OLB8	–	–	–	X	–
Chitinophagales	*Parafilimonas*	–	–	X	–	–
Chitinophagales	*Parasegetibacter*	–	–	X	–	–
Chitinophagales	*Phaeodactylibacter*	–	X	X	X	X
Chitinophagales	*Portibacter*	–	X	–	–	–
Chitinophagales	*Puia*	–	–	X	–	X
Chitinophagales	*Rurimicrobium*	X	X	X	–	–
Chitinophagales	*Sediminibacterium*	X	X	–	X	–
Chitinophagales	*Taibaiella*	–	–	–	–	X
Chitinophagales	*Terrimonas*	X	X	X	X	–
Chlorobiales	*Chlorobium*	–	X	–	–	X
Chlorobiales	*Chloroherpeton*	–	–	–	–	X
Chlorobiales	*Prosthecochloris*	–	–	–	–	X
Chloroflexales	*Candidatus* Chloroploca	–	X	X	–	X
Chloroflexales	*Candidatus* Chlorothrix	–	X	X	X	X
Chloroflexales	*Chloronema*	X	X	X	–	X
Chloroflexales	*Herpetosiphon*	–	–	X	–	–
Chloroflexales	*Oscillochloris*	–	X	X	–	X
Chloroflexales	*Roseiflexus*	–	–	–	–	X
Christensenellales	Christensenellaceae R‐7 group	–	X	X	X	X
Chromatiales	*Candidatus* Thiobios	–	–	–	–	X
Chromatiales	*Chromatium*	–	X	–	X	–
Chromatiales	*Halochromatium*	–	X	–	–	X
Chromatiales	*Lamprocystis*	–	X	X	–	–
Chromatiales	*Thiocapsa*	X	X	X	–	X
Chromatiales	*Thiocystis*	–	X	X	X	X
Chromatiales	*Thiodictyon*	–	X	–	–	–
Chromatiales	*Thiohalocapsa*	–	–	–	–	X
Chromatiales	*Thiophaeococcus*	–	–	–	–	X
Chromatiales	*Thiorhodococcus*	–	–	–	–	X
Chthoniobacterales	*Candidatus* Udaeobacter	–	X	–	X	–
Chthoniobacterales	*Candidatus* Xiphinematobacter	–	X	X	–	–
Chthoniobacterales	*Chthoniobacter*	–	X	X	X	X
Chthoniobacterales	FukuN18 freshwater group	–	–	X	–	X
Chthoniobacterales	LD29	–	X	X	–	X
Chthoniobacterales	*Terrimicrobium*	X	X	X	X	X
Chthonomonadales	*Chthonomonas*	–	X	–	X	–
Cloacimonadales	LNR A2‐18	–	X	–	–	X
Clostridiales	*Candidatus* Arthromitus	–	X	–	–	–
Clostridiales	*Clostridium* sensu stricto 1	–	X	X	X	X
Clostridiales	*Clostridium* sensu stricto 11	–	X	X	–	X
Clostridiales	*Clostridium* sensu stricto 12	–	X	X	X	–
Clostridiales	*Clostridium* sensu stricto 13	–	X	X	X	–
Clostridiales	*Clostridium* sensu stricto 16	–	–	–	X	–
Clostridiales	*Clostridium* sensu stricto 3	–	–	X	–	–
Clostridiales	*Clostridium* sensu stricto 5	–	X	–	–	–
Clostridiales	*Clostridium* sensu stricto 9	–	X	X	X	–
Clostridiales	*Clostridium*	–	X	X	–	–
Clostridiales	*Fonticella*	–	X	X	X	–
Clostridiales	*Oxobacter*	–	X	X	–	–
Clostridiales	*Proteiniclasticum*	–	–	X	X	–
Competibacterales	*Candidatus* Competibacter	–	X	X	X	X
Competibacterales	*Candidatus* Contendobacter	X	X	X	–	–
Corynebacteriales	*Corynebacterium*	–	–	–	–	–
Corynebacteriales	*Mycobacterium*	–	X	X	X	X
Coxiellales	*Coxiella*	X	X	X	X	X
Cyanobacteriales	*Aliterella*	X	–	–	–	X
Cyanobacteriales	*Annamia* HOs24	–	X	–	–	X
Cyanobacteriales	*Arthrospira* PCC‐7345	–	–	–	–	X
Cyanobacteriales	*Calothrix* PCC‐6303	X	X	–	–	X
Cyanobacteriales	*Chroococcidiopsis* PCC 7203	–	–	X	–	X
Cyanobacteriales	*Cyanothece* PCC‐7424	–	–	–	–	X
Cyanobacteriales	*Ewamiania* TS0513	–	X	–	–	–
Cyanobacteriales	*Geitlerinema* LD9	X	–	X	–	–
Cyanobacteriales	*Geitlerinema* PCC‐9228	–	X	–	–	X
Cyanobacteriales	*Geminocystis* PCC‐6308	–	X	X	X	–
Cyanobacteriales	*Gloeocapsa* PCC‐7428	–	–	–	–	X
Cyanobacteriales	*Gloeocapsa*	–	X	X	X	X
Cyanobacteriales	*Kamptonema* PCC‐6407	–	X	X	X	–
Cyanobacteriales	*Lyngbya* PCC‐7419	–	X	–	–	–
Cyanobacteriales	*Mastigocladopsis* PCC‐10914	–	–	–	–	X
Cyanobacteriales	*Merismopedia* 0BB39S01	–	X	X	–	–
Cyanobacteriales	*Microcoleus*	X	X	X	X	X
Cyanobacteriales	*Microcystis* PCC‐7914	–	–	–	X	X
Cyanobacteriales	*Myxosarcina* GI1	–	–	–	–	X
Cyanobacteriales	*Nostoc* PCC‐7107	–	X	–	–	X
Cyanobacteriales	*Nostoc* PCC‐73102	–	–	X	–	–
Cyanobacteriales	*Nostoc* PCC‐7524	–	–	–	–	X
Cyanobacteriales	*Oscillatoria* PCC‐10802	–	X	–	X	–
Cyanobacteriales	*Oscillatoria* PCC‐6304	–	X	X	–	–
Cyanobacteriales	*Phormidium* IAM M‐71	–	X	X	–	–
Cyanobacteriales	*Planktothricoides* SR001	–	X	X	–	–
Cyanobacteriales	*Planktothrix* NIVA‐CYA 15	X	X	X	X	X
Cyanobacteriales	*Pleurocapsa* PCC‐7319	–	–	–	–	X
Cyanobacteriales	*Scytonema* VB‐61278	–	–	–	–	X
Cyanobacteriales	*Snowella* 0TU37S04	–	–	–	X	–
Cyanobacteriales	*Spirulina* CCC Snake P‐Y‐85	–	–	–	–	X
Cyanobacteriales	*Spirulina* PCC‐6313	–	X	–	X	–
Cyanobacteriales	*Synechocystis* CCALA 700	–	X	X	–	X
Cyanobacteriales	*Synechocystis* PCC‐6803	–	–	X	–	X
Cyanobacteriales	*Synechocystis* SAG 90.79	–	–	–	–	X
Cyanobacteriales	*Trichodesmium* IMS101	–	X	X	–	–
Cyanobacteriales	*Tychonema* CCAP 1459‐11B	X	X	X	X	–
Cytophagales	*Adhaeribacter*	–	X	X	–	–
Cytophagales	*Algoriphagus*	–	X	X	–	X
Cytophagales	*Arcicella*	–	–	X	X	–
Cytophagales	*Bernardetia*	X	X	X	–	–
Cytophagales	*Candidatus* Amoebophilus	X	X	X	X	X
Cytophagales	*Chryseolinea*	X	X	X	X	X
Cytophagales	*Cytophaga*	X	X	X	X	–
Cytophagales	*Dyadobacter*	X	X	X	–	–
Cytophagales	*Ekhidna*	–	–	X	–	X
Cytophagales	*Emticicia*	X	X	X	–	X
Cytophagales	*Fibrella*	X	X	X	–	–
Cytophagales	*Flectobacillus*	–	–	X	–	X
Cytophagales	*Flexibacter*	–	X	X	–	X
Cytophagales	*Fluviimonas*	–	–	–	–	X
Cytophagales	*Hassallia*	–	X	X	X	–
Cytophagales	*Hymenobacter*	–	–	X	–	X
Cytophagales	*Imperialibacter*	–	–	–	–	X
Cytophagales	*Lacihabitans*	–	X	X	X	X
Cytophagales	*Larkinella*	X	–	X	–	X
Cytophagales	*Mariniradius*	–	–	–	–	X
Cytophagales	*Marinoscillum*	–	–	–	–	X
Cytophagales	*Ohtaekwangia*	–	–	X	–	–
Cytophagales	OLB12	X	X	X	X	X
Cytophagales	*Pseudarcicella*	–	X	X	–	–
Cytophagales	*Raineya*	–	X	X	–	X
Cytophagales	*Rapidithrix*	–	–	–	–	X
Cytophagales	*Rhabdobacter*	–	–	X	–	X
Cytophagales	*Rhodocytophaga*	–	–	X	–	X
Cytophagales	*Roseivirga*	–	–	–	–	X
Cytophagales	*Rudanella*	–	X	–	–	–
Cytophagales	*Runella*	X	X	X	X	X
Cytophagales	*Spirosoma*	X	X	X	–	X
Cytophagales	*Sporocytophaga*	X	X	X	–	–
Cytophagales	*Thermoflexibacter*	X	–	X	–	–
Defferrisomatales	*Deferrisoma*	–	X	X	–	–
Defluviicoccales	*Defluviicoccus*	X	X	X	X	X
Dehalococcoidales	*Dehalogenimonas*	–	X	X	–	–
Deinococcales	*Deinococcus*	X	X	X	–	X
Deinococcales	*Truepera*	X	–	X	–	X
Desulfarculales	*Desulfocarbo*	–	–	–	–	X
Desulfatiglandales	*Desulfatiglans*	–	X	X	X	X
Desulfitobacteriales	*Dehalobacter*	–	–	–	–	X
Desulfitobacteriales	*Desulfosporosinus*	X	X	X	X	–
Desulfobaccales	*Desulfobacca*	X	X	X	X	X
Desulfobacterales	*Desulfatirhabdium*	X	X	X	X	X
Desulfobacterales	*Desulfatitalea*	–	X	X	–	X
Desulfobacterales	*Desulfobacter*	–	–	X	–	X
Desulfobacterales	*Desulfobacterium*	–	X	X	X	–
Desulfobacterales	*Desulfococcaceae*	–	X	X	X	–
Desulfobacterales	*Desulfococcus*	–	X	X	–	X
Desulfobacterales	*Desulfonema*	X	X	X	X	–
Desulfobacterales	*Desulforegula*	–	–	–	–	X
Desulfobacterales	*Desulfosarcina*	–	–	–	–	X
Desulfobacterales	*Incertae Sedis*	–	X	X	X	X
Desulfobacterales	LCP‐80	–	X	X	–	X
Desulfobacterales	SEEP‐SRB1	X	X	X	X	X
Desulfobacterales	Sva0081 sediment group	–	X	X	X	X
Desulfobulbales	[*Desulfobacterium*] catecholicum group	X	X	X	X	X
Desulfobulbales	*Candidatus* Electronema	–	–	–	X	–
Desulfobulbales	*Desulfobulbus*	X	X	X	X	X
Desulfobulbales	*Desulfocapsa*	X	X	X	X	–
Desulfobulbales	*Desulfopila*	–	X	–	–	X
Desulfobulbales	*Desulfurivibrio*	X	X	–	–	X
Desulfobulbales	MSBL7	X	X	–	–	X
Desulfomonilales	*Desulfomonile*	X	X	X	X	X
Desulfotomaculales	*Desulfofarcimen*	–	X	X	–	–
Desulfotomaculales	*Desulfurispora*	–	X	–	–	–
Desulfovibrionales	*Desulfocurvus*	–	X	–	–	–
Desulfovibrionales	*Desulfomicrobium*	–	X	X	X	X
Desulfovibrionales	*Desulfovibrio*	X	X	X	X	X
Desulfuromonadales	*Desulfuromonas*	–	X	–	–	–
Diplorickettsiales	*Aquicella*	–	X	X	X	–
Diplorickettsiales	*Rickettsiella*	–	X	X	–	X
Dissulfuribacterales	SEEP‐SRB2	–	–	X	–	–
Dongiales	*Dongia*	–	X	X	X	–
Ectothiorhodospirales	*Thiohalophilus*	–	–	–	–	X
Elsterales	*Elstera*	X	X	X	–	–
Elusimicrobiales	*Elusimicrobium*	–	–	–	–	X
Endomicrobiales	*Endomicrobium*	X	X	X	X	X
Enterobacterales	*Aeromonas*	X	X	X	X	X
Enterobacterales	*Alishewanella*	–	–	–	–	X
Enterobacterales	*Arsenophonus*	–	–	–	–	X
Enterobacterales	*Gallaecimonas*	–	–	–	–	X
Enterobacterales	*Hafnia‐Obesumbacterium*	–	X	–	–	–
Enterobacterales	*Klebsiella*	X	–	–	–	X
Enterobacterales	*Mangrovibacter*	–	–	–	–	X
Enterobacterales	*Pantoea*	–	–	X	–	X
Enterobacterales	*Pectobacterium*	–	–	X	–	–
Enterobacterales	*Plesiomonas*	–	X	–	–	–
Enterobacterales	*Pseudobowmanella*	–	–	–	–	X
Enterobacterales	*Rheinheimera*	X	X	X	X	X
Enterobacterales	*Shewanella*	–	X	X	–	X
Enterobacterales	*Tolumonas*	–	–	–	–	X
Enterobacterales	*Vibrio*	–	–	–	–	X
Enterobacterales	*Yersinia*	–	–	X	X	–
Erysipelotrichales	*Breznakia*	–	X	–	–	–
Erysipelotrichales	*Erysipelatoclostridium*	–	–	X	–	–
Erysipelotrichales	*Erysipelothrix*	X	X	X	X	–
Erysipelotrichales	*Turicibacter*	–	–	–	–	X
Erysipelotrichales	ZOR0006	–	X	X	–	–
Eubacteriales	*Acetobacterium*	–	X	X	X	–
Eubacteriales	*Alkalibacter*	–	X	–	–	–
Eubacteriales	*Anaerofustis*	–	X	X	–	–
Exiguobacterales	*Exiguobacterium*	–	X	X	–	X
Ferrovibrionales	*Ferrovibrio*	–	–	–	–	X
Fibrobacterales	*Fibrobacter*	–	–	–	–	X
Fibrobacterales	Possible genus 04	–	X	X	–	–
Fibrobacterales	Possible genus 06	–	X	X	X	X
Flavobacteriales	*Actibacter*	–	X	X	X	X
Flavobacteriales	*Apibacter*	–	–	X	–	–
Flavobacteriales	*Aureicoccus*	–	X	X	–	–
Flavobacteriales	*Chryseobacterium*	–	X	X	–	X
Flavobacteriales	*Cloacibacterium*	–	–	–	–	X
Flavobacteriales	*Crocinitomix*	–	X	X	X	X
Flavobacteriales	*Cryomorpha*	–	X	–	X	X
Flavobacteriales	*Flavobacterium*	X	X	X	X	X
Flavobacteriales	*Fluviicola*	X	X	X	X	X
Flavobacteriales	*Gramella*	–	–	–	–	–
Flavobacteriales	*Hoppeia*	–	–	–	–	–
Flavobacteriales	*Lutibacter*	–	–	X	–	–
Flavobacteriales	*Maritimimonas*	X	X	X	X	–
Flavobacteriales	*Neptunitalea*	–	–	–	–	X
Flavobacteriales	*Ornithobacterium*	–	–	–	–	X
Flavobacteriales	*Robiginitalea*	–	–	–	–	X
Flavobacteriales	*Schleiferia*	–	–	–	–	X
Flavobacteriales	*Vicingus*	–	X	–	–	–
Frankiales	*Candidatus* Planktophila	–	X	–	–	–
Frankiales	*Longivirga*	–	X	X	X	–
Frankiales	*Nakamurella*	–	–	X	–	–
Frankiales	*Sporichthya*	–	–	X	X	–
Fusobacteriales	*Cetobacterium*	–	X	X	–	X
Fusobacteriales	*Fusobacterium*	–	X	–	–	–
Fusobacteriales	*Hypnocyclicus*	–	X	X	X	X
Gaiellales	*Gaiella*	–	X	X	X	–
Gammaproteobacteria Incertae Sedis	*Acidibacter*	X	X	X	–	X
Gammaproteobacteria Incertae Sedis	*Candidatus* Berkiella	X	X	X	X	X
Gammaproteobacteria Incertae Sedis	*Candidatus* Endonucleariobacter	–	X	X	–	–
Gammaproteobacteria Incertae Sedis	*Candidatus* Ovatusbacter	X	X	X	X	X
Gemmatales	*Fimbriiglobus*	X	X	X	X	X
Gemmatales	*Gemmata*	X	X	X	X	X
Gemmatales	*Telmatocola*	–	X	–	X	X
Gemmatales	*Tuwongella*	X	X	X	X	X
Gemmatales	*Zavarzinella*	–	X	X	X	–
Gemmatimonadales	*Gemmatimonas*	–	X	X	X	X
Geobacterales	*Citrifermentans*	–	–	X	–	–
Geobacterales	*Geobacter*	X	X	X	X	–
Geobacterales	*Pseudopelobacter*	X	X	–	X	–
Geobacterales	*Trichlorobacter*	X	–	X	X	X
GIF9	SCGC‐AB‐539‐J10	–	X	X	–	–
Gloeobacterales	*Gloeobacter* PCC‐7421	X	X	X	X	–
Haliangiales	*Haliangium*	X	X	X	X	X
Halothiobacillales	*Halothiobacillus*	X	–	–	–	X
Halothiobacillales	*Thiovirga*	X	X	X	X	X
Holophagales	*Geothrix*	–	–	–	X	–
Holosporales	*Candidatus* Bealeia	–	–	–	–	–
Holosporales	*Candidatus* Paraholospora	–	–	X	–	–
Hydrogenedentiales	YC‐ZSS‐LKJ63	–	–	X	X	X
Ignavibacteriales	*Ignavibacterium*	–	X	X	X	X
Ignavibacteriales	IheB3‐7	X	X	X	X	X
Isosphaerales	*Aquisphaera*	–	X	X	–	–
Isosphaerales	*Isosphaera*	–	X	X	–	X
Isosphaerales	*Paludisphaera*	X	–	–	–	–
Isosphaerales	*Tundrisphaera*	X	X	X	–	–
Kiloniellales	*Tistlia*	–	–	–	–	X
Kiritimatiellales	MSBL3	X	X	X	X	X
Kiritimatiellales	R76‐B128	X	X	X	–	X
Ktedonobacterales	1959‐1	–	X	X	–	–
Lachnospirales	*Anaerocolumna*	–	X	–	–	–
Lachnospirales	*Cellulosilyticum*	–	X	X	X	X
Lachnospirales	Defluviitaleaceae UCG‐011	–	X	X	X	X
Lachnospirales	*Epulopiscium*	–	X	X	–	X
Lachnospirales	*Herbinix*	–	X	–	–	–
Lachnospirales	Lachnospiraceae NK4A136 group	–	–	X	–	–
Lachnospirales	Lachnospiraceae UCG‐010	–	–	–	–	X
Lachnospirales	*Lachnotalea*	–	X	–	–	–
Lachnospirales	*Mobilitalea*	–	X	–	–	–
Lachnospirales	*Natranaerovirga*	–	–	–	–	X
Lachnospirales	*Tyzzerella*	–	X	X	–	X
Lachnospirales	XBB1006	–	–	X	X	–
Lactobacillales	*Catellicoccus*	–	X	X	–	–
Lactobacillales	*Enterococcus*	–	–	X	–	X
Lactobacillales	*Floricoccus*	–	–	–	–	X
Lactobacillales	*Lactobacillus*	–	–	X	–	–
Lactobacillales	*Lactococcus*	–	X	X	X	X
Lactobacillales	*Leuconostoc*	–	–	–	–	X
Lactobacillales	*Ligilactobacillus*	–	–	X	–	–
Lactobacillales	*Trichococcus*	–	X	X	X	–
Latescibacterales	*Candidatus* Latescibacter	–	X	X	X	X
Legionellales	*Legionella*	X	X	X	X	X
Leptolyngbyales	*Arthronema* SAG 12.89	–	X	–	–	X
Leptolyngbyales	*Calothrix* KVSF5	–	X	–	X	–
Leptolyngbyales	*Chamaesiphon* PCC‐7430	–	–	X	–	–
Leptolyngbyales	JSC‐12	–	X	–	–	–
Leptolyngbyales	*Leptolyngbya* ANT.L52.2	X	X	X	–	X
Leptolyngbyales	*Leptolyngbya* ANT.L67.1	X	X	–	–	–
Leptolyngbyales	*Leptolyngbya* BN43	–	X	–	–	–
Leptolyngbyales	*Leptolyngbya* FYG	–	–	X	–	–
Leptolyngbyales	*Leptolyngbya* SAG 2411	–	X	–	X	–
Leptolyngbyales	*Limnolyngbya* CHAB4449	X	X	X	–	X
Leptolyngbyales	*Oscillatoria* SAG 1459‐8	–	X	–	–	–
Leptolyngbyales	*Phormidesmis* ANT.L52.6	X	–	–	–	–
Leptolyngbyales	*Phormidium* CYN64	–	X	–	X	–
Leptolyngbyales	TG‐45	–	X	–	–	–
Leptospirales	*Leptospira*	X	X	X	X	X
Leptospirales	RBG‐16‐49‐21	X	X	X	X	X
Leptospirales	*Turneriella*	X	X	X	X	X
Leptospirillales	*Leptospirillum*	–	X	X	–	–
Methanobacteriales	*Methanobacterium*	–	X	X	X	X
Methanocellales	Rice Cluster I	–	–	–	X	–
Methanofastidiosales	*Candidatus* Methanofastidiosum	–	–	–	X	–
Methanomicrobiales	*Methanocorpusculum*	–	–	–	–	X
Methanomicrobiales	*Methanoregula*	–	X	–	X	–
Methanomicrobiales	*Methanosphaerula*	–	–	X	–	–
Methanosarciniales	*Methanolobus*	–	–	X	–	X
Methanosarciniales	*Methanomethylovorans*	–	–	X	–	X
Methanosarciniales	*Methanosaeta*	–	X	X	X	–
Methanosarciniales	*Methanosarcina*	–	X	X	X	–
Methylococcales	*Candidatus* Methylospira	–	X	–	–	–
Methylococcales	*Crenothrix*	X	X	X	X	–
Methylococcales	*Methylobacter*	–	X	–	X	–
Methylococcales	*Methylocaldum*	–	X	X	–	X
Methylococcales	*Methyloglobulus*	–	X	X	–	–
Methylococcales	*Methylomonas*	X	X	–	–	–
Methylococcales	*Methylovulum*	–	X	X	–	–
Methylococcales	pLW‐20	–	X	–	X	–
Methylomirabilales	*Candidatus* Methylomirabilis	–	X	–	–	–
Methylomirabilales	Sh765B‐TzT‐35	–	X	X	X	–
Methylomirabilales	wb1‐A12	–	X	X	–	–
Micrococcales	*Agromyces*	–	–	X	–	–
Micrococcales	*Aquipuribacter*	–	X	X	–	–
Micrococcales	*Candidatus* Aquiluna	–	X	–	–	–
Micrococcales	*Candidatus* Planktoluna	–	–	–	–	–
Micrococcales	*Cellulomonas*	–	–	X	–	–
Micrococcales	*Chryseoglobus*	–	–	X	–	–
Micrococcales	*Cryobacterium*	–	X	X	–	–
Micrococcales	*Demequina*	–	X	–	–	–
Micrococcales	*Galbitalea*	–	X	X	–	–
Micrococcales	*Gryllotalpicola*	–	X	–	–	–
Micrococcales	*Leifsonia*	–	–	X	–	–
Micrococcales	*Leucobacter*	–	–	–	X	–
Micrococcales	*Microbacterium*	–	X	X	–	X
Micrococcales	MWH‐Ta3	–	–	–	–	–
Micrococcales	*Oryzihumus*	–	–	X	–	–
Micrococcales	*Pseudarthrobacter*	–	X	X	–	–
Micrococcales	*Rhodoluna*	–	X	–	–	–
Micromonosporales	*Catellatospora*	–	X	X	–	–
Micromonosporales	*Luedemannella*	–	X	–	–	–
Micromonosporales	*Micromonospora*	–	–	X	–	–
Micromonosporales	*Virgisporangium*	–	–	X	–	–
Micropepsales	*Micropepsis*	–	–	–	–	X
Micropepsales	*Rhizomicrobium*	–	–	X	–	–
Microtrichales	CL500‐29 marine group	–	X	X	X	–
Microtrichales	*Iamia*	–	X	X	X	–
Microtrichales	*Ilumatobacter*	X	X	X	X	X
Microtrichales	IMCC26207	–	X	X	X	X
Moduliflexales	Candidatus *Moduliflexus*	–	X	X	X	–
Monoglobales	*Monoglobus*	X	–	–	X	–
Mycoplasmatales	*Candidatus* Bacilloplasma	–	X	X	–	X
Myxococcales	*Anaeromyxobacter*	–	X	X	X	–
Myxococcales	*Archangium*	–	–	X	–	–
Myxococcales	KD3‐10	X	X	X	–	X
Myxococcales	*Myxococcus*	–	–	X	–	–
Myxococcales	P3OB‐42	X	X	X	X	X
Nannocystales	*Nannocystis*	–	X	X	X	X
Nannocystales	*Pseudenhygromyxa*	–	–	–	–	X
Nitrosococcales	CI75cm.2.12	–	X	X	–	–
Nitrosococcales	wb1‐P19	–	X	X	–	–
Nitrosopumilales	*Nitrosarchaeum*	–	X	X	–	–
Nitrososphaerales	*Candidatus* Nitrocosmicus	–	–	X	–	–
Nitrososphaerales	*Candidatus* Nitrososphaera	–	–	X	–	–
Nitrospirales	*Nitrospira*	–	X	X	X	–
Obscuribacterales	*Candidatus* Obscuribacter	X	X	X	X	–
Oligoflexales	*Oligoflexus*	–	X	X	X	X
Oligoflexales	*Pseudobacteriovorax*	–	–	–	–	X
Oligosphaerales	SBZC‐1223	–	X	–	–	–
Omnitrophales	*Candidatus* Omnitrophus	X	X	X	X	X
Opitutales	*Alterococcus*	–	X	X	X	X
Opitutales	*Cephaloticoccus*	–	X	–	–	–
Opitutales	*Cerasicoccus*	–	X	X	–	X
Opitutales	*Diplosphaera*	–	–	–	–	X
Opitutales	IMCC26134	X	X	X	–	X
Opitutales	*Lacunisphaera*	–	X	X	X	X
Opitutales	*Lentimonas*	–	–	–	–	X
Opitutales	*Opitutus*	–	X	X	X	–
Opitutales	*Pelagicoccus*	–	–	–	–	X
Opitutales	*Puniceicoccus*	–	X	X	–	X
Opitutales	Verruc‐01	–	X	X	–	X
Oscillospirales	*Anaerobacterium*	–	X	X	–	–
Oscillospirales	*Candidatus Soleaferrea*	–	–	–	–	X
Oscillospirales	*Caproiciproducens*	–	X	X	X	–
Oscillospirales	*Colidextribacter*	–	–	–	–	X
Oscillospirales	*Ercella*	–	X	X	X	–
Oscillospirales	*Faecalibacterium*	–	–	X	–	–
Oscillospirales	HN‐HF0106	–	X	X	X	–
Oscillospirales	*Incertae Sedis*	–	–	–	–	X
Oscillospirales	*Incertae Sedis*	–	X	–	–	X
Oscillospirales	*Intestinimonas*	–	–	–	X	–
Oscillospirales	NK4A214 group	–	X	–	X	–
Oscillospirales	*Paludicola*	–	–	–	X	–
Oscillospirales	*Pseudobacteroides*	–	–	X	–	–
Oscillospirales	*Ruminiclostridium*	–	X	X	X	X
Oscillospirales	*Ruminococcus*	–	X	X	X	X
Oscillospirales	*Saccharofermentans*	–	X	X	X	–
Oscillospirales	*Sporobacter*	–	X	X	–	X
Oscillospirales	UCG‐005	–	–	–	–	X
Oscillospirales	UCG‐012	–	X	X	–	–
Oxyphotobacteria Incertae Sedis	*Leptolyngbya* EcFYyyy‐00	–	X	–	–	–
Oxyphotobacteria Incertae Sedis	*Pseudanabaena* NgrPSln22	–	–	–	–	X
Paenibacillales	*Ammoniphilus*	–	–	X	–	–
Paenibacillales	*Cohnella*	–	–	X	–	–
Paenibacillales	*Paenibacillus*	–	X	X	–	X
Paenibacillales	*Saccharibacillus*	–	–	X	–	–
Paracaedibacterales	*Candidatus* Captivus	X	X	X	X	–
Paracaedibacterales	*Candidatus* Finniella	–	–	X	X	–
Paracaedibacterales	*Candidatus* Paracaedibacter	X	X	X	–	X
Pedosphaerales	ADurb.Bin063‐1	–	X	X	X	–
Pedosphaerales	ADurb.Bin118	–	X	X	X	X
Pedosphaerales	DEV008	–	X	X	X	X
Pedosphaerales	DEV114	–	–	–	X	–
Pedosphaerales	Ellin516	–	–	X	–	–
Pedosphaerales	*Oikopleura*	–	X	X	X	X
Pedosphaerales	*Pedosphaera*	–	X	–	X	–
Pedosphaerales	SCGC AAA164‐E04	–	–	–	–	X
Pedosphaerales	SH3‐11	X	X	X	X	X
Peptostreptococcales‐Tissierellales	[Eubacterium] brachy group	–	X	X	X	–
Peptostreptococcales‐Tissierellales	*Acidaminobacter*	X	X	X	X	X
Peptostreptococcales‐Tissierellales	*Anaerovorax*	X	X	X	X	X
Peptostreptococcales‐Tissierellales	*Fusibacter*	X	X	X	X	X
Peptostreptococcales‐Tissierellales	*Paeniclostridium*	–	X	–	–	X
Peptostreptococcales‐Tissierellales	*Paraclostridium*	–	X	X	–	–
Peptostreptococcales‐Tissierellales	*Proteocatella*	–	X	X	–	–
Peptostreptococcales‐Tissierellales	*Romboutsia*	–	X	X	–	X
Peptostreptococcales‐Tissierellales	*Sedimentibacter*	–	–	X	X	X
Peptostreptococcales‐Tissierellales	*Terrisporobacter*	–	–	–	–	X
Peptostreptococcales‐Tissierellales	*Tissierella*	–	–	X	–	–
Petrotogales	SC103	–	–	–	X	–
Phormidesmiales	*Leptolyngbya* PCC‐6406	–	–	–	–	X
Phormidesmiales	MBIC10086	–	–	–	–	X
Phormidesmiales	*Nodosilinea* PCC‐7104	–	X	X	X	X
Phormidesmiales	*Phormidium* MBIC10003	–	X	–	–	X
Phycisphaerales	AKYG587	–	X	X	X	–
Phycisphaerales	CL500‐3	–	X	–	X	X
Phycisphaerales	*Phycisphaera*	–	X	X	X	X
Phycisphaerales	SM1A02	X	X	X	X	X
Phycisphaerales	*Urania*‐1B‐19 marine sediment group	–	X	–	–	X
Pirellulales	*Blastopirellula*	–	X	X	X	X
Pirellulales	*Bythopirellula*	–	X	X	–	X
Pirellulales	*Candidatus* Anammoximicrobium	–	X	X	X	X
Pirellulales	Pir1 lineage	–	X	–	–	–
Pirellulales	Pir2 lineage	–	X	X	–	–
Pirellulales	Pir3 lineage	–	X	X	X	X
Pirellulales	Pir4 lineage	X	X	X	X	X
Pirellulales	*Pirellula*	X	X	X	X	X
Pirellulales	*Rhodopirellula*	X	X	X	X	X
Pirellulales	*Rubripirellula*	–	–	–	–	X
Piscirickettsiales	*Candidatus* Endoecteinascidia	–	X	–	–	–
Planctomycetales	*Planctomicrobium*	–	X	X	X	X
Planctomycetales	*Planctopirus*	X	X	X	X	X
Planctomycetales	*Rubinisphaera*	X	X	X	X	X
Planctomycetales	*Schlesneria*	X	X	X	X	–
Planctomycetales	SH‐PL14	–	X	X	X	–
Polyangiales	*Minicystis*	–	–	X	–	–
Polyangiales	*Pajaroellobacter*	X	X	X	X	X
Polyangiales	*Phaselicystis*	X	X	X	X	X
Polyangiales	*Polyangium*	–	X	X	–	–
Polyangiales	*Sandaracinus*	X	X	X	–	X
Propionibacteriales	*Cutibacterium*	X	–	X	–	–
Propionibacteriales	*Marmoricola*	–	–	X	–	–
Propionibacteriales	*Microlunatus*	–	X	X	–	–
Propionibacteriales	*Nocardioides*	–	X	X	X	–
Propionibacteriales	*Propionicicella*	–	X	–	–	–
Pseudanabaenales	*Pseudanabaena* PCC‐6802	–	X	–	–	–
Pseudanabaenales	*Pseudanabaena* PCC‐7429	–	X	X	X	–
Pseudanabaenales	*Synechococcus* PCC‐7502	–	X	–	–	–
Pseudomonadales	[*Agitococcus*] lubricus group	–	–	X	–	–
Pseudomonadales	*Acinetobacter*	–	X	X	–	–
Pseudomonadales	*Alkanindiges*	–	X	X	X	–
Pseudomonadales	*Balneatrix*	–	–	–	–	X
Pseudomonadales	BD1‐7 clade	–	X	–	X	–
Pseudomonadales	*Cellvibrio*	–	X	–	–	X
Pseudomonadales	*Chromatocurvus*	–	–	–	X	–
Pseudomonadales	*Enhydrobacter*	–	–	X	–	–
Pseudomonadales	*Fluviicoccus*	–	X	–	–	–
Pseudomonadales	*Hahella*	–	X	X	X	X
Pseudomonadales	*Halioglobus*	–	–	X	X	–
Pseudomonadales	*Luminiphilus*	–	–	–	–	X
Pseudomonadales	*Microbulbifer*	–	–	–	–	X
Pseudomonadales	*Oceanobacter*	–	–	–	–	X
Pseudomonadales	OM60(NOR5) clade	X	X	X	X	X
Pseudomonadales	*Pseudohongiella*	–	–	–	X	X
Pseudomonadales	*Pseudomonas*	X	X	X	–	X
Pseudomonadales	*Psychrobacter*	–	X	X	–	–
Pseudomonadales	*Reinekea*	–	–	–	–	X
Pseudonocardiales	*Actinomycetospora*	–	–	–	–	X
Pseudonocardiales	*Pseudonocardia*	–	–	X	–	X
Pyrinomonadales	RB41	–	X	X	–	–
Reyranellales	*Reyranella*	–	X	X	X	–
Rhizobiales	1174‐901‐12	–	–	–	–	X
Rhizobiales	*Allorhizobium‐Neorhizobium‐Pararhizobium‐Rhizobium*	X	X	X	–	X
Rhizobiales	alphaI cluster	–	X	X	X	–
Rhizobiales	*Aureimonas*	–	–	X	–	–
Rhizobiales	*Bauldia*	–	X	X	–	–
Rhizobiales	*Bosea*	X	X	X	–	–
Rhizobiales	*Bradyrhizobium*	–	X	X	–	–
Rhizobiales	*Chthonobacter*	–	X	X	–	–
Rhizobiales	*Cohaesibacter*	–	–	–	–	X
Rhizobiales	*Devosia*	X	X	X	X	–
Rhizobiales	*Ensifer*	–	–	X	–	–
Rhizobiales	*Filomicrobium*	X	X	X	–	X
Rhizobiales	*Hoeflea*	X	–	–	–	–
Rhizobiales	*Hyphomicrobium*	X	X	X	X	X
Rhizobiales	*Kaistia*	–	X	–	–	–
Rhizobiales	*Mesorhizobium*	–	X	X	–	–
Rhizobiales	*Methylobacterium‐Methylorubrum*	–	–	X	X	X
Rhizobiales	*Methyloceanibacter*	–	–	–	–	X
Rhizobiales	*Methylocystis*	–	X	–	–	–
Rhizobiales	*Microvirga*	–	–	X	–	–
Rhizobiales	*Nitratireductor*	–	X	X	X	X
Rhizobiales	*Nordella*	–	X	X	X	X
Rhizobiales	*Pedomicrobium*	–	X	X	–	–
Rhizobiales	*Phreatobacter*	X	X	X	X	X
Rhizobiales	*Pleomorphomonas*	–	X	X	–	X
Rhizobiales	*Prosthecomicrobium*	–	X	X	–	–
Rhizobiales	*Pseudolabrys*	–	X	X	–	–
Rhizobiales	*Pseudorhizobium*	–	–	–	–	X
Rhizobiales	*Pseudorhodoplanes*	–	X	X	X	–
Rhizobiales	*Pseudoxanthobacter*	X	–	X	–	–
Rhizobiales	*Rhodomicrobium*	–	X	X	–	–
Rhizobiales	*Rhodoplanes*	–	X	–	–	–
Rhizobiales	*Shinella*	–	X	X	–	–
Rhizobiales	*Tardiphaga*	–	X	–	–	–
Rhizobiales	*Xanthobacter*	–	–	X	–	–
Rhodobacterales	*Actibacterium*	–	–	–	–	X
Rhodobacterales	*Amaricoccus*	–	X	X	X	X
Rhodobacterales	*Cereibacter*	X	X	X	–	–
Rhodobacterales	*Defluviimonas*	–	–	–	X	–
Rhodobacterales	*Flavimaricola*	–	X	X	X	–
Rhodobacterales	*Gemmobacter*	X	X	X	X	X
Rhodobacterales	*Limibaculum*	–	X	X	–	X
Rhodobacterales	*Oceanicella*	–	X	–	–	X
Rhodobacterales	*Paracoccus*	X	–	X	–	–
Rhodobacterales	*Planktotalea*	–	–	–	–	X
Rhodobacterales	*Pseudorhodobacter*	X	X	X	X	X
Rhodobacterales	*Rhodobacter*	–	X	X	X	–
Rhodobacterales	*Rhodovulum*	–	X	–	–	X
Rhodobacterales	*Roseibaca*	–	–	–	–	X
Rhodobacterales	*Roseobacter* clade CHAB‐I‐5 lineage	–	–	X	–	–
Rhodobacterales	*Rubribacterium*	–	X	X	–	X
Rhodobacterales	*Rubrimonas*	–	–	–	–	X
Rhodobacterales	*Tabrizicola*	X	X	X	X	X
Rhodobacterales	*Thioclava*	X	–	–	–	–
Rhodobacterales	*Tropicimonas*	–	X	X	–	X
Rhodobacterales	*Yoonia‐Loktanella*	–	X	X	–	–
Rhodospirillales	*Candidatus* Riegeria	–	X	–	–	–
Rhodospirillales	*Pararhodospirillum*	–	–	–	–	X
Rhodospirillales	*Rhodospirillum*	–	–	X	–	–
Rhodothermales	*Rubrivirga*	–	X	–	–	–
Rickettsiales	*Candidatus* Cryptoprodotis	–	X	–	–	–
Rickettsiales	*Candidatus* Megaira	X	X	X	X	X
Rickettsiales	*Candidatus* Xenohaliotis	–	–	X	–	–
Rickettsiales	MD3‐55	–	–	–	X	–
Rickettsiales	*Rickettsia*	–	–	X	–	X
Salinisphaerales	*Nevskia*	–	–	–	–	X
SBR1031	OLB13	–	X	X	–	X
SBR1031	OLB15	–	X	X	–	–
Silvanigrellales	*Silvanigrella*	–	X	X	–	–
Solibacterales	*Candidatus* Solibacter	–	X	X	–	–
Solirubrobacterales	*Conexibacter*	–	X	X	X	–
Solirubrobacterales	*Parviterribacter*	–	X	X	X	–
Solirubrobacterales	*Solirubrobacter*	–	X	X	–	–
Sphingobacteriales	*Arcticibacter*	–	–	X	–	–
Sphingobacteriales	*Lentimicrobium*	X	X	X	X	X
Sphingobacteriales	*Mucilaginibacter*	–	–	–	–	X
Sphingobacteriales	*Pedobacter*	–	X	X	–	X
Sphingobacteriales	*Solitalea*	–	X	X	–	–
Sphingobacteriales	*Sphingobacterium*	–	–	X	–	–
Sphingomonadales	*Altererythrobacter*	X	X	X	–	–
Sphingomonadales	*Blastomonas*	X	X	X	–	X
Sphingomonadales	DSSF69	–	X	–	–	–
Sphingomonadales	*Erythrobacter*	X	X	X	–	X
Sphingomonadales	*Novosphingobium*	–	X	X	X	X
Sphingomonadales	*Polymorphobacter*	X	X	X	X	–
Sphingomonadales	*Porphyrobacter*	X	X	X	X	X
Sphingomonadales	*Qipengyuania*	–	–	X	–	–
Sphingomonadales	*Rhizorhapis*	–	–	X	X	–
Sphingomonadales	*Sandaracinobacter*	–	X	X	X	X
Sphingomonadales	*Sandarakinorhabdus*	–	X	–	X	X
Sphingomonadales	*Sphingobium*	–	–	–	–	X
Sphingomonadales	*Sphingomicrobium*	–	X	–	–	–
Sphingomonadales	*Sphingomonas*	X	–	X	–	–
Sphingomonadales	*Sphingopyxis*	X	X	X	–	–
Sphingomonadales	*Sphingorhabdus*	X	X	X	X	–
Sphingomonadales	*Sphingosinicella*	–	–	–	X	–
Spirochaetales	GWE2‐31‐10	–	X	X	X	X
Spirochaetales	M2PT2‐76 termite group	–	X	X	X	X
Spirochaetales	*Salinispira*	X	X	–	X	X
Spirochaetales	*Sediminispirochaeta*	–	X	–	X	X
Spirochaetales	*Sphaerochaeta*	–	–	X	X	–
Spirochaetales	*Spirochaeta 2*	X	X	X	X	X
Spirochaetales	*Spirochaeta*	X	X	X	X	X
Spirochaetales	*Treponema*	X	X	X	X	X
Staphylococcales	*Macrococcus*	–	X	–	–	–
Staphylococcales	*Staphylococcus*	–	X	X	–	X
Steroidobacterales	*Steroidobacter*	–	X	X	–	X
Steroidobacterales	*Woeseia*	–	X	X	X	–
Streptomycetales	*Allostreptomyces*	–	–	X	–	–
Streptomycetales	*Streptomyces*	–	X	X	–	–
Streptosporangiales	*Actinocorallia*	–	X	–	–	–
Streptosporangiales	*Nonomuraea*	–	–	X	–	–
Streptosporangiales	*Thermocatellispora*	–	X	–	–	–
Sumerlaeales	*Sumerlaea*	X	X	X	X	X
Symbiobacteriales	*Symbiobacterium*	–	X	X	–	–
Synechococcales	*Cyanobium* PCC‐6307	X	X	X	X	X
Synechococcales	*Limnothrix*	–	X	X	–	–
Synechococcales	*Prochlorothrix* PCC‐9006	–	X	X	–	–
Synechococcales	*Schizothrix* LEGE 07164	–	X	X	–	–
Synechococcales	*Synechococcus* MBIC10613	–	–	–	X	X
Synergistales	JGI‐0000079‐D21	–	–	X	–	–
Syntrophales	*Smithella*	–	–	X	X	–
Syntrophales	*Syntrophus*	–	X	X	X	X
Syntrophobacterales	*Desulfovirga*	–	X	–	–	–
Syntrophobacterales	*Syntrophobacter*	–	X	X	–	X
Syntrophomonadales	*Syntrophomonas*	–	–	X	–	–
Syntrophorhabdales	*Syntrophorhabdus*	–	X	X	X	–
Tepidisphaerales	*Tepidisphaera*	–	X	X	X	–
Thermincolales	*Thermincola*	–	X	X	–	–
Thermoactinomycetales	*Geothermomicrobium*	–	X	–	–	–
Thermoactinomycetales	*Melghirimyces*	–	X	–	–	–
Thermoactinomycetales	*Pasteuria*	–	X	X	–	X
Thermoactinomycetales	*Shimazuella*	–	X	–	–	–
Thermoactinomycetales	*Thermoflavimicrobium*	–	X	–	–	–
Thermoanaerobaculales	Subgroup 10	X	X	X	X	X
Thermoanaerobaculales	Subgroup 23	–	X	–	X	X
Thermoanaerobaculales	*Thermoanaerobaculum*	X	X	X	X	X
Thermoanaerobaculales	TPD‐58	–	X	X	X	–
Thiomicrospirales	*Hydrogenovibrio*	–	–	–	–	X
Thiomicrospirales	*Thiomicrorhabdus*	–	X	X	X	–
Thiotrichales	*Candidatus* Navis	–	–	–	–	X
Thiotrichales	*Thiothrix*	X	X	X	X	X
Tistrellales	*Candidatus* Alysiosphaera	X	X	X	X	X
Vampirovibrionales	*Vampirovibrio*	–	X	X	–	–
Veillonellales‐Selenomonadales	*Anaeromusa‐Anaeroarcus*	–	–	X	–	–
Veillonellales‐Selenomonadales	*Anaerosinus*	–	X	X	–	–
Veillonellales‐Selenomonadales	*Anaerospora*	–	X	X	–	–
Veillonellales‐Selenomonadales	*Pectinatus*	–	–	–	–	X
Veillonellales‐Selenomonadales	*Pelosinus*	–	X	X	X	X
Veillonellales‐Selenomonadales	*Propionispira*	–	–	X	–	X
Veillonellales‐Selenomonadales	*Sporomusa*	–	X	–	–	–
Veillonellales‐Selenomonadales	*Zymophilus*	–	X	–	–	–
Verrucomicrobiales	DBS1	–	–	X	–	–
Verrucomicrobiales	*Haloferula*	–	–	–	–	X
Verrucomicrobiales	*Luteolibacter*	X	X	X	X	X
Verrucomicrobiales	*Prosthecobacter*	–	X	–	X	X
Verrucomicrobiales	*Roseibacillus*	–	–	X	–	X
Verrucomicrobiales	*Roseimicrobium*	–	X	X	–	–
Verrucomicrobiales	*Rubritalea*	–	–	X	X	–
Verrucomicrobiales	*Verrucomicrobium*	–	X	X	X	–
Vicinamibacterales	*Luteitalea*	–	X	X	X	–
Vicinamibacterales	*Vicinamibacter*	–	X	X	–	–
Woesearchaeales	AR15	–	X	X	X	X
Xanthomonadales	*Ahniella*	–	X	X	X	X
Xanthomonadales	*Aquimonas*	X	X	X	–	X
Xanthomonadales	*Arenimonas*	X	X	X	X	–
Xanthomonadales	*Chiayiivirga*	–	–	–	–	–
Xanthomonadales	*Dokdonella*	X	–	–	X	–
Xanthomonadales	*Dyella*	–	–	–	–	X
Xanthomonadales	*Luteimonas*	X	X	X	–	–
Xanthomonadales	*Lysobacter*	–	–	X	–	–
Xanthomonadales	*Pseudoxanthomonas*	–	–	X	–	–
Xanthomonadales	*Rhodanobacter*	X	–	–	–	–
Xanthomonadales	*Silanimonas*	X	X	X	X	X
Xanthomonadales	*Stenotrophomonas*	–	–	X	–	X
Xanthomonadales	*Tahibacter*	X	–	X	–	–
Xanthomonadales	*Thermomonas*	X	X	X	–	–
Xanthomonadales	*Xanthomonas*	–	–	–	–	–

Abbreviations: ECB, El Cajon Bay; FTN, Alpena Fountain; GSS, Great Sulfur Spring; MIS, Middle Island Sinkhole; OAK, Florida Oak Spring.

**TABLE 3:** Diatom genera present (X) or absent (−) in each site.

Genus	FTN	ECB	GSS	MIS	OAK
*Achnanthes*	–	–	–	–	X
*Achnanthidium*	X	X	X	X	–
*Actinoptychus*	X	–	X	–	–
*Adlafia*	–	X	X	–	–
*Amphora*	X	X	X	X	X
*Arcocellulus*	X	–	X	–	–
*Bacillaria*	X	X	X	–	–
*Berkeleya*	–	–	–	X	–
*Brachysira*	X	X	X	X	X
*Caloneis*	–	X	X	X	–
*Campylodiscus*	X	X	X	–	–
*Cocconeis*	–	X	X	X	X
*Coronia*	–	X	X	–	–
*Craspedostauros*	–	–	X	–	–
*Craticula*	X	X	X	X	X
*Ctenophora*	–	X	–	–	–
*Cylindrotheca*	–	–	X	–	–
*Cymbella*	X	X	X	X	–
*Cymbopleura*	–	X	–	X	–
*Denticula*	–	–	–	X	–
*Diatoma*	–	X	–	X	–
*Dimeregramma*	–	–	X	–	–
*Diploneis*	X	X	X	X	X
*Discostella*	–	–	–	X	–
*Ellerbeckia*	–	–	–	X	–
*Encyonema*	–	X	X	X	–
*Encyonopsis*	X	X	X	X	–
*Entomoneis*	X	X	X	X	X
*Envekadea*	–	X	X	X	X
*Epithemia*	X	X	X	X	X
*Eunotia*	X	X	–	X	–
*Fallacia*	–	–	X	–	–
*Fistulifera*	–	–	X	–	–
*Fragilaria*	X	X	X	X	X
*Gedaniella*	X	X	X	–	–
*Geissleria*	–	–	–	X	–
*Gomphonema*	X	X	X	X	X
*Grammatophora*	–	–	–	X	–
*Gyrosigma*	X	–	X	–	–
*Halamphora*	X	X	X	–	X
*Hantzschia*	–	–	X	–	X
*Haslea*	X	–	X	–	–
*Hippodonta*	–	X	X	–	–
*Hyalosynedra*	–	–	–	–	X
*Iconella*	–	–	–	X	–
*Luticola*	–	–	X	–	–
*Mastogloia*	X	X	–	–	X
*Mayamaea*	–	–	X	–	–
*Melosira*	–	X	X	X	–
*Meridion*	–	X	–	–	–
*Minidiscus*	X	–	X	–	–
*Minutocellus*	X	X	X	–	–
*Nanofrustulum*	X	–	X	–	–
*Navicula*	X	X	X	X	X
*Neidium*	–	X	X	X	–
*Nitzschia*	X	X	X	X	X
*Opephora*	–	–	X	–	–
*Pantocsekiella*	–	–	–	X	–
*Paralia*	X	X	X	–	–
*Parlibellus*	–	–	–	X	–
*Pinnularia*	–	X	X	X	X
*Planothidium*	X	X	X	X	X
*Pleurosigma*	–	–	X	–	X
*Psammodictyon*	X	X	X	–	–
*Psammothidium*	–	–	–	X	–
*Pseudofalcula*	–	–	–	–	X
*Rossithidium*	–	X	–	X	–
*Sellaphora*	X	X	X	X	–
*Seminavis*	–	–	–	–	X
*Serratifera*	–	–	–	–	X
*Simonsenia*	–	–	X	–	–
*Stauroforma*	X	X	X	–	–
*Stauroneis*	–	X	X	X	–
*Staurosira*	X	X	X	X	X
*Stephanodiscus*	–	–	–	X	–
*Surirella*	–	–	X	X	–
*Tabellaria*	–	–	–	X	–
*Tabularia*	–	–	–	X	–
*Terpsinoe*	–	X	X	–	–
*Thalassiosira*	X	X	X	–	–
*Tryblionella*	–	–	X	X	X
*Ulnaria*	X	X	X	X	–

Abbreviations: ECB, El Cajon Bay; FTN, Alpena Fountain; GSS, Great Sulfur Spring; MIS, Middle Island Sinkhole; OAK, Florida Oak Spring.

### 3.4 Diversity

**FIGURE 2:**
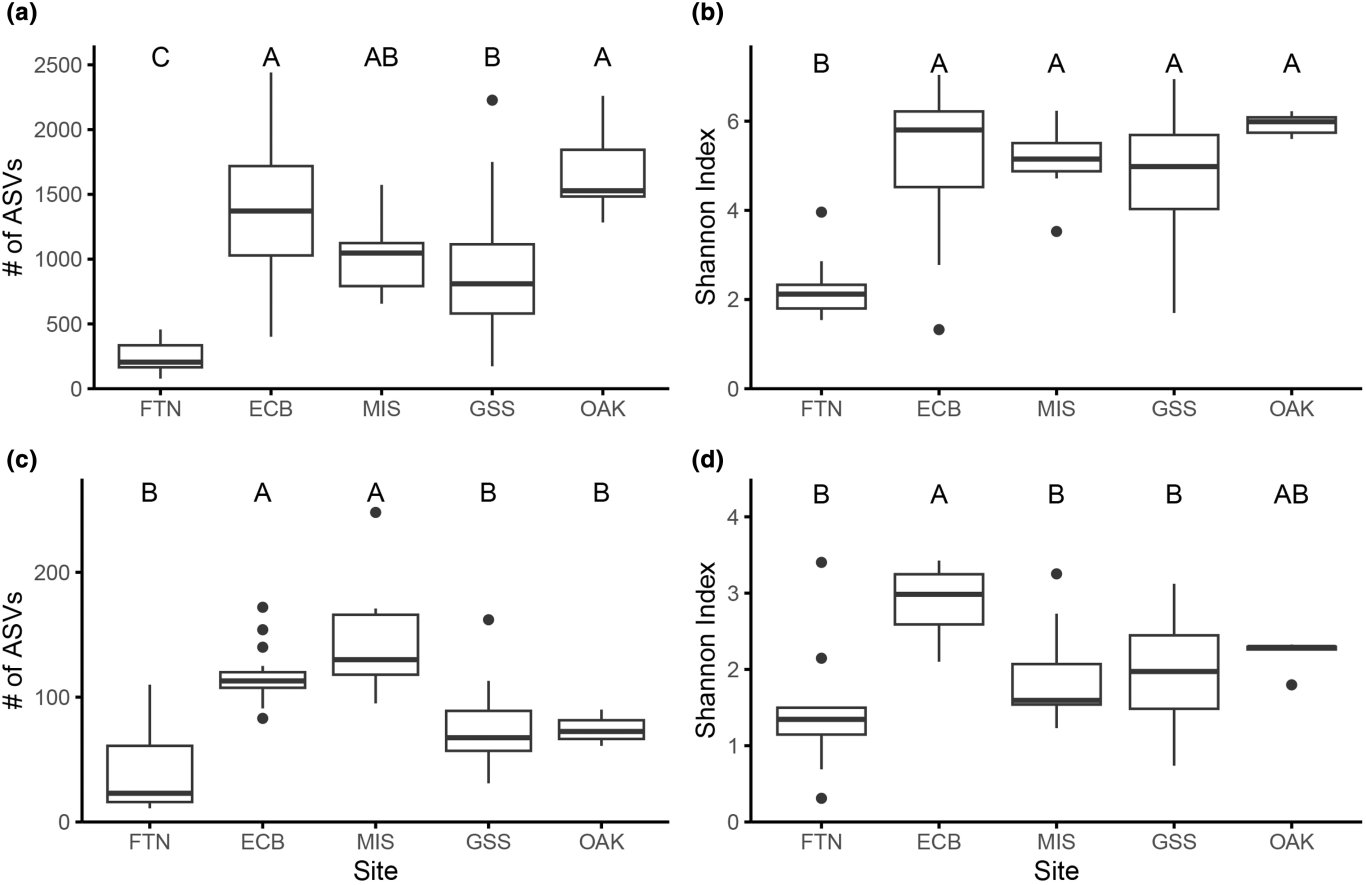
Boxplots representing ASV richness and Shannon alpha diversity metrics for the 16S (a, b) and *rbc*L (c, d) datasets at each site. Sites sharing a capital letter are not significantly different as determined by one‐way ANOVAs with Tukey post‐hoc tests (*p* < .05). Thick black lines within the box represent median values, boxes represent the interquartile range, and whiskers and points represent the range, with points outside the whiskers representing outliers (values over or under 1.5 times the interquartile range). ECB, El Cajon Bay; FTN, Alpena Fountain; GSS, Great Sulfur Spring; MIS, Middle Island Sinkhole; OAK, Florida Oak Spring.

For the 16S dataset, FTN had significantly lower observed ($\bar{x}$ = 242, *H*_4_ = 32.4, *p* < .001) and Shannon ($\bar{x}$ = 2.3, *F*_4,65_ = 10.9, *p <* .001) diversity than the other sites (Figure 2a,b). The 16S observed diversity was intermediate for GSS ($\bar{x}$ = 929) and highest for ECB ($\bar{x}$ = 1395), MIS ($\bar{x}$ = 1029), and OAK ($\bar{x}$ = 1670). The 16S Shannon diversity was similar for ECB ($\bar{x}$ = 5.27), MIS ($\bar{x}$ = 5.12), GSS ($\bar{x}$ = 4.74), and OAK ($\bar{x}$ = 5.93).

For the *rbc*L dataset, MIS ($\bar{x}$ = 144) and ECB ($\bar{x}$ = 116) had the highest observed diversity (*H*_4_ = 49.1, *p <* .001), followed by GSS ($\bar{x}$ = 70.7) and OAK ($\bar{x}$ = 74.1), and then FTN ($\bar{x}$ = 42.2) with the lowest observed diversity (Figure 2c,d). ECB had the highest Shannon diversity ($\bar{x}$ = 2.91, *H*_4_ = 35.5, *p <* .001), with OAK having an intermediate value ($\bar{x}$ = 2.21) and all of the other sites (FTN ($\bar{x}$ = 1.46), MIS ($\bar{x}$ = 1.91), and GSS ($\bar{x}$ = 1.97)) having similar lower values.

Overall, observed alpha diversity was an order of magnitude higher for the 16S than the *rbc*L dataset (Figure 2). For both 16S and *rbc*L, FTN had relatively low diversity and ECB had relatively high diversity. At OAK, the relatively high diversity of the 16S observed and Shannon diversity were contrasted by low *rbc*L diversity.

**FIGURE 3:**
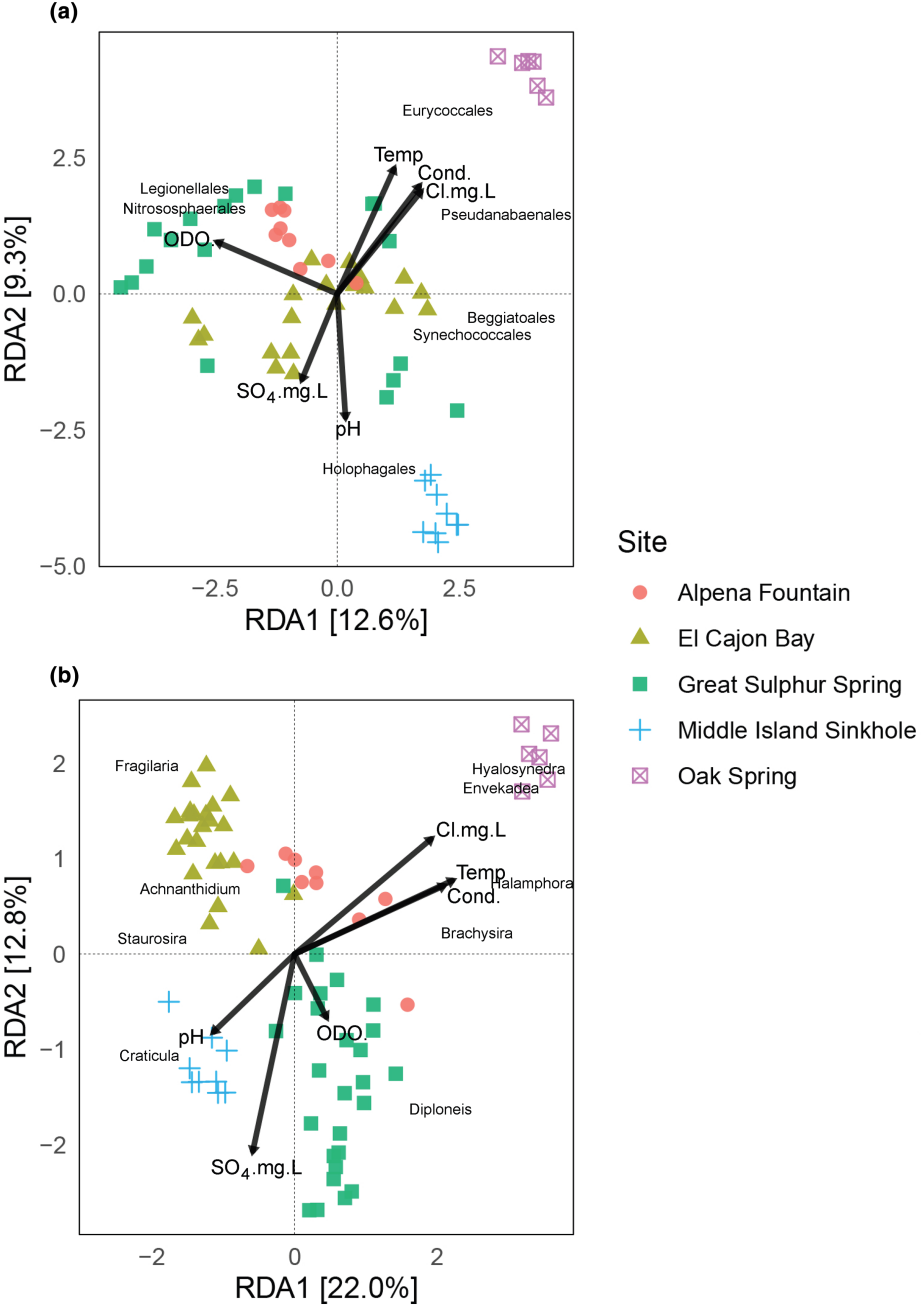
Redundancy analysis (RDA) ordinations showing relationships between environmental variables and taxa explained by the axes for the 16S (a) and *rbc*L (b) datasets, respectively. Variables include percent dissolved oxygen (ODO.), temperature (Temp), specific conductivity (Cond.), pH, sulfate (SO_4_.mg.L), and chloride (Cl.mg.L).

Beta diversity between sites was significantly different for both 16S (*F*_4,65_ = 6.72, *p* < .001) and *rbc*L markers (*F*_4,78_ = 22.93, *p* < .001). A pairwise PERMANOVA post‐hoc test revealed that all sites differed from each other for each marker (*p* < .001 for all pairwise comparisons). These site differences are presented in the clustering of samples by site in RDA ordinations (Figure 3).

### 3.5 Factors contributing to community differences

All environmental variables measured were significant and included in RDA ordinations (Figure 3). The first two axes of the 16S RDA explained only 21.9% of the total variance. The 16S RDA1 axis was explained by gradients of chloride (Cl.mg.L), conductivity (Cond.), and temperature (Temp), all of which were high in OAK. Dissolved oxygen (ODO.) also presented a gradient on this axis for 16S, with lower values associated with MIS and higher values with GSS. The 16S ordination RDA2 axis was strongly correlated with pH, with MIS samples associating with high values. High pH was associated with the Holophagales (*r*(68) = .49, *p* < .001), and low dissolved oxygen was associated with the Beggiatoales (*r*(68) = −.67, *p* < .001) and the Synechococcales (*r*(68) = −.70, *p* < .001).

Slightly more variance was explained by the first two axes of the *rbc*L RDA (34.8%). The *rbc*L RDA1 axis was also explained by a gradient of chloride (Cl.mg.L), conductivity (Cond.), and temperature (Temp), and OAK samples were associated with high values of these variables, along with the marine taxa *Hyalosynedra* and *Envekadea*. The *rbc*L RDA2 axis was explained by dissolved oxygen (ODO.), sulfate (SO_4_.mg.L), and pH gradients. High dissolved oxygen and sulfate differentiated the GSS samples on the *rbc*L ordination from ECB, MIS, and FTN. *Diploneis* showed a correlation with high sulfate concentrations (*r*(84) = .74, *p* < .001), and higher pH values were correlated with *Craticula* (*r*(84) = .357, *p* = .003). The environmental variables for both 16S and *rbc*L indicate that increased pH may be contributing to the unique microbial community in MIS.

Mantel tests found that the environmental variables measured were significantly correlated (*p* < .05) with the community distance matrices for both the 16S and *rbc*L datasets (Table 4). A matrix of all environmental variables measured (AllEnv) was tested against a matrix of geographic distances to determine the importance of biogeography and local conditions to the changes in the community matrix. For both markers, both the geographic distance and the environmental variables showed significant correlation with the community matrix, with the geographic correlations (16S: *r* = .5331, *rbc*L: *r* = .6022) being slightly stronger than the environmental correlations (16S: *r* = .3894, *rbc*L: *r* = .4621).

**TABLE 4:** Mantel test results for environmental variables and geographic distance.

Mantel test results				
	*rbc*L		16S	
	*r*	*p*	r	*p*
Geography	.6022	.0001	.5331	.0001
AllEnv	.4621	.0001	.3894	.0001
Cl	.7946	.0001	.7016	.0001
Cond	.5054	.0001	.3976	.001
Si	.4488	.0001	.4231	.0001
Temp	.414	.0001	.4801	.0001
pH	.3551	.0001	.1617	.0044
SO_4_	.236	.0002	.2631	.0001
DO%	.1036	.0223	.1431	.0069

*Note*: *R* values indicate the strength of correlation with changes in the community dissimilarity matrix (0 = not correlated; 1 = strongly correlated). Where *p*‐values <.05, changes in the variable are correlated with changes in the community dissimilarity matrix. All variables were significant.

Abbreviations: AllEnv, matrix containing all environmental variables measured; Cl, chloride; Cond, conductivity; DO%, percent dissolved oxygen; Si, silica concentration; Temp, temperature; SO_4_, sulfate concentration.

### 3.6 Community composition

**FIGURE 4:**
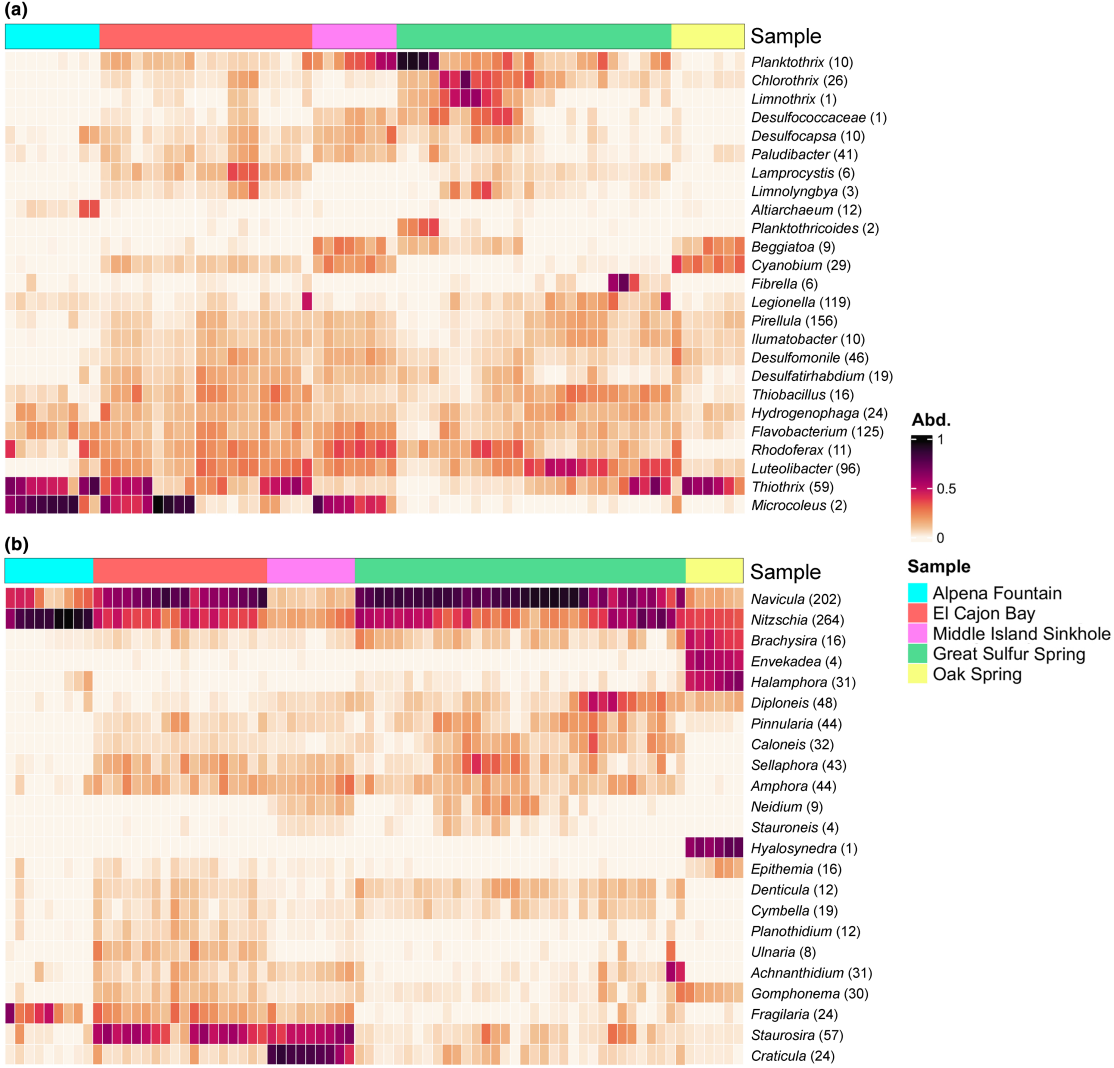
Heatmaps displaying bacterial/archaeal genera (a, 16S) and diatom genera (b, *rbc*L) that composed of the highest relative abundances of samples from each site. Color across the top of each plot indicates site, and color of each box indicates Hellinger‐transformed relative abundance. The number of ASVs assigned to each group is listed in parentheses next to each taxon.

The most abundant taxa at each site are shown by Hellinger‐transformed relative abundances in heatmaps (Figure 4). For the cyanobacteria, *Planktothrix* and *Limnothrix* dominated GSS samples, while FTN, ECB, and MIS were composed primarily of *Microcoleus. Thiothrix*, a genus of sulfur‐oxidizing bacteria, was abundant in some samples from each of the sites, except for MIS. The MIS bacterial community was more diverse and less dominated by a single genus with *Beggiatoa* and *Rhodoferax* at higher abundance. For the diatom community, a variety of genera contributed to the abundance in each sample. Most samples were dominated by the speciose *Navicula* and *Nitzschia*, except for MIS. MIS contained primarily *Craticula* and *Staurosira*, whereas OAK was dominated by *Brachysira, Halamphora*, and the marine genera *Hyalosynedra* and *Envekadea*.

## 4 DISCUSSION

### 4.1 Water parameters

Despite a common groundwater aquifer source providing a constant flow of compositionally similar water (Snider et al., 2017), conditions at MIS, ECB, and FTN differed in temperature, pH, and chloride concentrations. A main driver of this habitat variety may be mixing with surface water, which is nonexistent at FTN, limited at MIS (Ruberg et al., 2008), and constant at ECB (Snider et al., 2017). Low‐oxygen, high‐sulfur conditions at these springs contrast with the surrounding lake waters bordering MIS, ECB, and GSS, where percent dissolved oxygen levels approach complete saturation and sulfate concentrations are below 40 mg/L (Biddanda et al., 2012; Haack et al., 2005). Percent dissolved oxygen of most samples approached or exceeded the threshold for hypoxia (30%, Steckbauer et al., 2011), while the values recorded from MIS and the GSS source approached anoxic conditions. While pH differed between MIS and GSS, these sites were similar in conductivity, percent dissolved oxygen, nitrate, and sulfate concentrations, presenting comparable unique conditions for mat communities at both locations. Despite these similarities, a direct comparison of these mat samples revealed significantly different microbial communities for both the 16S and *rbc*L markers (PERMANOVA, *p* < .001). pH differed between MIS and GSS. Depth is another factor that differentiates these two sites (MIS = 23 m; GSS = 13 m), with light availability for photoautotrophs more limited in MIS than in GSS. Similar water parameters were found at GSS to those recorded previously, except for pH, which was 6.4 in Chaudhary et al. (2009) and 7.35 in our study. OAK differed from other sites in temperature, conductivity, and chloride, factors related to its warmer climate and proximity to the Atlantic Ocean. Analyses of the main salts contributing to high chloride concentrations (e.g., NaCl, KCl, MgCl) would provide more insight into the causes of high chloride in the groundwater at these sites. OAK had low dissolved oxygen (similar to the Michigan sites) and had sulfate concentrations similar to the Alpena, Michigan sites (FTN, ECB, and MIS).

### 4.2 Diversity

Our metabarcoding approach revealed high levels of bacterial diversity in MIS. Kinsman‐Costello et al. (2017) also reported high bacterial diversity from MIS, but differences in sample processing and data analyses prevent a direct comparison of alpha diversity. Our study also revealed a diverse microbial community in GSS, which had not been previously investigated with high‐throughput sequencing techniques but had been documented to contain cyanobacteria, sulfur‐metabolizing bacteria, and Archaea using clone libraries (Chaudhary et al., 2009). Additionally, few explorations of eukaryotic diversity have occurred at these sites (except Nold, Pangborn, et al., 2010), and our study presents the first targeted survey of diatom diversity at MIS, ECB, FTN, GSS, and OAK. Distinct bacterial and diatom communities were found at each site, despite the shared groundwater sources and geographic proximity between some sites (e.g., <20 km between FTN, ECB, and MIS). These sulfur spring sites presented a range of habitats. ECB and GSS have increased habitat complexity, which has been correlated with increased diversity of freshwater benthic microbial communities (Levi et al., 2017; Singer et al., 2010). Higher nitrate concentrations at ECB could also contribute to more algal taxa inhabiting the site. However, low nutrients in the water measured at MIS may not result in limitation for microbes, as the sediment beneath the microbial mat is known to accumulate organic material and promote nutrient flux to surface mats (Kinsman‐Costello et al., 2017). Measurements of flux at the sediment–water interface would be useful to compare nutrient availability as a contribution to microbial diversity at other sites in the future. The *rbc*L dataset showed significantly higher Shannon diversity at ECB and OAK than other sites, while GSS showed intermediate diversity values. Influence from surrounding surface waters at sites with higher levels of surface mixing such as ECB could also lead to higher diversity values due to increased dispersal of free‐floating microbes. Microbes from surrounding waters were undoubtably collected within our microbial mat samples, but our plankton tows allowed us to eliminate some of this suspended community from our analyses. Plankton tow samples were composed mainly of *Lindavia* (Malik & Saros, 2016) and *Cyclotella* (Saros & Anderson, 2015), taxa that are commonly found in the water column, justifying their removal from the analyses. While dispersal abilities of suspended microbes present an unavoidable issue when characterizing benthic microbial community composition, using plankton tow sampling to eliminate taxa from further analyses of benthic communities can increase accuracy, particularly for groups such as diatoms where growth habits are well established. However, since planktonic and benthic algal communities influence each other (Stevenson et al., 1996), the amount of settled cells in a benthic community could have impact on its structure and function. The diversity of diatom taxa we found within these microbial mat communities presents the need for more research on eukaryotic mat community members, and the roles they may play in these mats.

### 4.3 Factors contributing to community differences

The stark difference between OAK mat communities and other sites was strongly associated with temperature, conductivity, and chloride concentrations. MIS was associated with higher pH than the other sites. The sites differentiated more distinctly by environmental variables in the *rbc*L dataset than 16S, indicating that these variables may influence diatom communities more strongly than bacteria. This could also be due to the increased taxonomic resolution we were able to use (order for 16S vs. genus for *rbc*L). The *rbc*L ordination showed positive dissolved oxygen and sulfate gradients associated with GSS that were not observed in the 16S ordination, suggesting that the bacterial communities at GSS may be influenced by variables that were not measured. These trends in community dissimilarity indicate that environmental variables may vary strongly in their influence on different members of mat communities (e.g., Lu et al., 2023).

Metabarcoding studies have been useful for exploring biogeographic patterns of diversity and taxonomy in bacteria (Varliero et al., 2023) and provide an opportunity to develop large datasets describing microbial communities that can be used with environmental and geographic variables to determine factors influencing these communities. Despite significant effects of the environmental variables driving the diatom and bacterial community composition of these microbial mats, the variables measured explained a low percentage of variance.

Geographic distance showed a significant correlation with differences in community composition in this study. Major barriers to dispersion exist between these isolated sites. While cosmopolitan species may travel through the Great Lakes, these isolated habitats are unlikely to be reached by microbes specializing in high‐sulfur, low‐oxygen conditions. Groundwater is a likely source for some of the microbes in these communities, particularly bacteria. Further studies of groundwater aquifer biodiversity, along with exploration of the evolutionary history of taxa in these isolated spring ecosystems, could help answer important questions about dispersal and its role in the microbial biogeography of these sites.

### 4.4 Community composition

As expected, cyanobacterial ASVs were abundant in the 16S‐generated community. Interestingly, macroscopically similar purple‐colored cyanobacterial mats observed in GSS, MIS, and ECB did not result in similar bacterial beta diversity. The Synechococcales and Pseudanabaenales (Cyanobacteria), along with the Beggiatoales (sulfur‐oxidizing Bacteria), were associated with low dissolved oxygen in the RDA ordination. The Holophagales, a rare and poorly described anaerobic group (Anderson et al., 2011), contributed to separation between the MIS and GSS sites. Despite being dominated by cyanobacteria, differences in other Bacteria and Archaea may drive significant differences in community composition in microbial mats. Our 16S primers amplified some archaeal taxa, including the ammonium‐oxidizing order Nitrososphaerales (Könneke et al., 2005), which was associated with high dissolved oxygen and GSS sites. While archaeal diversity is poorly understood (Adam et al., 2017) and universal 16S primers may be unable to detect many Archaea (Eloe‐Fadrosh et al., 2016), these primers may allow for limited quantification of archaeal communities (Fadeev et al., 2021). Development of Archaea‐specific primers for metabarcoding may be required to better understand their diversity and functional role at these sites.

The *rbc*L marker revealed a diverse array of diatom taxa with high taxonomic resolution. This study adds to previous metabarcoding research that has used the *rbc*L marker to successfully characterize algal communities (e.g., Fawley et al., 2021; Pérez‐Burillo et al., 2022; Wolf & Vis, 2019). Culturing diatoms from our samples proved to be an important and successful method to improve the accuracy of taxonomic assignment. For the Michigan sites, almost half the reads generated (42.6%) were represented in our culturing efforts, increasing our confidence in the taxonomy assignment for these diatom communities. In total, 119 taxonomy assignments conflicted with species‐level assignments in Diat.barcode, suggesting that regional differences between our sites and those used to create the reference database (i.e., Michigan and Florida vs primarily European taxa) could lead to discrepancies, and further stressing the value of incorporating a culture‐based DNA reference library into metabarcoding studies. Species‐level taxonomy assignment is difficult even with sufficient reference information due to cryptic and unresolved species complexes, which can be found in ubiquitous groups such as *Fragilaria* (Van de Vijver et al., 2022) and *Nitzschia* (Rimet et al., 2014). Additionally, we found morphologically distinct *Cymbella* isolates share identical *rbc*L sequences for the metabarcoding region, suggesting the short (312 bp) *rbc*L region may be too conserved for species‐level identification in some genera. While genus‐level identification can provide sufficient resolution for accurate biomonitoring (Rimet & Bouchez, 2012), species‐level identification is a component of many biomonitoring programs because species within the same genus may differ widely in responses to water quality (e.g., Ponader & Potapova, 2007), especially for large, diverse genera with many species (Lowe, 1974) such as *Navicula* (Reavie et al., 2006) and *Nitzschia* (Hamsher et al., 2004).

Our culturing efforts yielded a wide variety of diatom genera on WC medium, but no taxa from OAK were cultured successfully, indicating a mismatch between our medium and site conditions that may be overcome with a more rigorous culturing effort. Cyanobacterial culturing success was limited mainly to *Anagnostidanema* and *Microcoleus* but still contributed valuable reference information for taxonomic assignment. A strategic culturing effort pairs well with a metabarcoding survey to characterize microbial communities and could be strengthened further if the use of longer marker regions is made possible by future sequencing technology.

Most of the dominant diatoms found in these microbial mat communities represented benthic, motile groups. Biddanda et al. (2023) noted that the mass vertical microbial migration of microbial mats occurs at a small scale but may have large impacts on metabolic processes in the mat. Motile diatoms may actively participate in this, such as *Craticula* optimizing nitrogen respiration in low light conditions (Merz et al., 2021). While the focus of most microbial mat research has remained on cyanobacteria due to their conspicuity and abundance, diatoms may also serve an important role. Future studies should investigate other motile diatoms in microbial mat communities to see if they may share this unique metabolic strategy, or partake in another.

Our study presents the first diatom surveys performed at these sites. GSS microbial mats were dominated by *Navicula oblonga*. This taxon may occupy a similar role in the mat community to *Craticula cuspidata* that dominate MIS, as both are motile taxa with similar autecology (Lowe, 1974). The presence and relatively large cell size (>100 μm) of *Navicula oblonga* in GSS samples could also contribute to an increased number of reads for each individual and overrepresent the abundance of *Navicula* in our analyses, an issue that may be resolved by developing correction factors for such taxa (Vasselon et al., 2018). *Nitzschia* were found in all site groups, and their role in microbial mat communities merits further investigation. Additionally, cryptic diversity within the *Nitzschia palea* species complex was noted in our cultured sequences (data not shown), and these isolated sites could provide further insight into the evolutionary history of this taxon. OAK diatom communities were dominated by *Hyalosynedra*, characterized as a benthic marine genus (Belando et al., 2018). This was surprising in a groundwater‐fed habitat isolated from marine surface waters, although conductivity and chloride measures suggested that water conditions at OAK could be considered brackish (Remane & Schlieper, 1971).

An issue with using DNA to characterize or explore algal communities is the persistence of DNA in water. Environmental DNA may persist long enough to be transported in the water column and consequently be detected at locations where the organism has not actually been present (Carraro et al., 2018; Shogren et al., 2018). Several studies show that eDNA persistence in water may reach 4 weeks, but most degradation occurs within the first few days (Collins et al., 2018; Lance et al., 2017; Strickler et al., 2015; Tsuji et al., 2017; Weltz et al., 2017). In contrast, eDNA in sediment and biofilms has been known to persist for longer time periods (Corinaldesi et al., 2005; Domaizon et al., 2017). While seasonal and geographic variations should be considered, 16S rRNA marker genes for *Bacteriodes* have been shown to persist in water for over a week when held at 10°C (Okabe & Shimazu, 2007). Logistical limitations due to difficult site access required MIS samples to remain at 10°C for 72 h. Aforementioned studies of aquatic degradation of DNA suggest that despite some sample processing limitations, our results should be considered reliable and reasonable sample processing times may be allowed for this type of study, with caution.

## 5 CONCLUSION

A multi‐marker metabarcoding analysis of microbial mats revealed diverse algal communities. Complex interactions between a variety of environmental factors and dispersal limitation appear to drive diversity in these isolated underwater habitats. Multi‐marker metabarcoding in combination with culturing presents a powerful tool for exploring algal diversity and the factors that may contribute to microbial community composition. Increased culturing efforts are recommended to contribute to reference information and strengthen the power of metabarcoding for future studies.

## AUTHOR CONTRIBUTIONS

**Davis Fray:** Conceptualization (equal); data curation (equal); formal analysis (equal); funding acquisition (supporting); investigation (equal); methodology (equal); project administration (equal); resources (supporting); software (equal); validation (equal); visualization (equal); writing – original draft (lead); writing – review and editing (equal). **Callahan A. McGovern:** Conceptualization (equal); data curation (equal); formal analysis (equal); investigation (equal); methodology (equal); project administration (equal); software (equal); validation (equal); visualization (equal); writing – original draft (equal); writing – review and editing (equal). **Dale A. Casamatta:** Conceptualization (equal); data curation (equal); formal analysis (equal); funding acquisition (equal); investigation (equal); methodology (equal); project administration (equal); resources (equal); software (equal); supervision (equal); validation (equal); visualization (equal); writing – original draft (equal); writing – review and editing (equal). **Bopaiah A. Biddanda:** Conceptualization (equal); data curation (equal); formal analysis (equal); funding acquisition (equal); investigation (equal); methodology (equal); project administration (equal); resources (equal); software (equal); supervision (equal); validation (equal); visualization (equal); writing – original draft (equal); writing – review and editing (equal). **Sarah E. Hamsher:** Conceptualization (equal); data curation (equal); formal analysis (equal); funding acquisition (equal); investigation (equal); methodology (equal); project administration (equal); resources (equal); software (equal); supervision (lead); validation (equal); visualization (equal); writing – original draft (equal); writing – review and editing (equal).

## CONFLICT OF INTEREST STATEMENT

The authors declare that they have no conflict of interest.

## Data Availability

Sequence data for this project are deposited in NCBI BioProject PRJNA1005976 (metabarcoding data), NCBI OR455849‐OR455891 (Sanger sequencing of diatom cultures), and NCBI MZ005297‐MZ005304, ON258648, OR582729‐OR582731 (Sanger sequencing of Cyanobacteria cultures). Associated metadata can be accessed through the National Science Foundation BCO‐DMO (10.26008/1912/bco‐dmo.911441.1, 10.26008/1912/bco‐dmo.911008.1, 10.26008/1912/bco‐dmo.911338.1) and R code for processing the raw metabarcoding data are available on Zenodo (10.5281/zenodo.10019983, and 10.5281/zenodo.10019989).

## References

[ece311162-bib-0001] Adam, P. S. , Borrel, G. , Brochier‐Armanet, C. , & Gribaldo, S. (2017). The growing tree of Archaea: New perspectives on their diversity, evolution and ecology. The ISME Journal, 11, 2407–2425. 28777382 10.1038/ismej.2017.122 PMC5649171

[ece311162-bib-0002] Allwood, A. C. (2016). Evidence of life in Earth's oldest rocks. Nature, 537, 500–501. 27580031 10.1038/nature19429

[ece311162-bib-0125] Alverson, A. J. , Jansen, R. K. , & Theriot, E. C. (2007). Bridging the Rubicon: Phylogenetic analysis reveals repeated colonizations of marine and fresh waters by thalassiosiroid diatoms. Molecular Phylogenetics and Evolution, 45, 193–210. 17553708 10.1016/j.ympev.2007.03.024

[ece311162-bib-0004] Anderson, M. J. , Crist, T. O. , Chase, J. M. , Vellend, M. , Inouye, B. D. , Freestone, A. L. , Sanders, N. J. , Cornell, H. V. , Comita, L. S. , Davies, K. F. , Harrison, S. P. , Kraft, N. J. B. , Stegen, J. C. , & Swenson, N. G. (2011). Navigating the multiple meanings of β diversity: A roadmap for the practicing ecologist. Ecology Letters, 14, 19–28. 21070562 10.1111/j.1461-0248.2010.01552.x

[ece311162-bib-0005] Antich, A. , Palacín, C. , Zarcero, J. , Wangensteen, O. S. , & Turon, X. (2022). Metabarcoding reveals high‐resolution biogeographical and metaphylogeographical patterns through marine barriers. Journal of Biogeography, 50, 515–527.

[ece311162-bib-0006] Apothéloz‐Perret‐Gentil, L. , Bouchez, A. , Cordier, T. , Cordonier, A. , Guéguen, J. , Rimet, F. , Vasselon, V. , & Pawlowski, J. (2021). Monitoring the ecological status of rivers with diatom eDNA metabarcoding: A comparison of taxonomic markers and analytical approaches for the inference of a molecular diatom index. Molecular Ecology, 30, 2959–2968. 32979002 10.1111/mec.15646 PMC8358953

[ece311162-bib-0007] Barnett, D. , Arts, I. , & Penders, J. (2021). microViz: An R package for microbiome data visualization and statistics. Journal of Open Source Software, 6, 3201.

[ece311162-bib-0008] Barrios, K. (2006). St. Marks River and Wakulla River Springs Inventory Leon and Wakulla counties. Northwest Florida Water Management District.

[ece311162-bib-0009] Bass Becking LGMB . (1934). Geobiologie of inleiding tot de milieukunde. W.P. Van Stockum & Zoon.

[ece311162-bib-0010] Belando, M. D. , Jiménez, J. F. , Marín, A. , & Aboal, M. (2018). Morphology and molecular phylogeny of *Hyalosynedra lanceolata* sp. nov. and an extended description of *Hyalosynedra* (Bacillariophyta). European Journal of Phycology, 53, 208–218.

[ece311162-bib-0011] Biddanda, B. , McMillan, A. , Long, S. , Snider, M. , & Weinke, A. (2015). Seeking sunlight: Rapid phototactic motility of filamentous mat‐forming cyanobacteria optimize photosynthesis and enhance carbon burial in Lake Huron's submerged sinkholes. Frontiers in Microbiology, 6, 930. 26441867 10.3389/fmicb.2015.00930 PMC4561352

[ece311162-bib-0012] Biddanda, B. A. , Coleman, D. F. , Johengen, T. H. , Ruberg, S. A. , Meadows, G. A. , Van Sumeren, H. W. , Rediske, R. R. , & Kendall, S. T. (2006). Exploration of a Submerged Sinkhole Ecosystem in Lake Huron. Ecosystems, 9, 828–842.

[ece311162-bib-0013] Biddanda, B. A. , Weinke, A. D. , & Stone, I. P. (2023). Extant mat microbes synchronize vertical migration to a diel tempo. Journal of Great Lakes Research, 49, 220–228.

[ece311162-bib-0131] Biddanda, B. , Nold, S. , Dick, G. , Kendall, S. , Vail, J. , & Ruberg, S. (2012). Rock, water, microbes: Sinkholes in Lake Huron are habitats for ancient microbial life. Nature Education Knowledge, 3, 13.

[ece311162-bib-0015] Burgsdorf, I. , Erwin, P. M. , López‐Legentil, S. , Cerrano, C. , Haber, M. , Frenk, S. , & Steindler, L. (2014). Biogeography rather than association with cyanobacteria structures symbiotic microbial communities in the marine sponge *Petrosia ficiformis* . Frontiers in Microbiology, 5, 529. 25346728 10.3389/fmicb.2014.00529 PMC4193313

[ece311162-bib-0016] Callahan, B. J. , McMurdie, P. J. , Rosen, M. J. , Han, A. W. , Johnson, A. J. A. , & Holmes, S. P. (2016). DADA2: High resolution sample inference from Illumina amplicon data. Nature Methods, 13, 581–583. 27214047 10.1038/nmeth.3869 PMC4927377

[ece311162-bib-0017] Canfield, D. E. , & Des Marais, D. J. (1993). Biogeochemical cycles of carbon, sulfur, and free oxygen in a microbial mat. Geochimica et Cosmochimica Acta, 57, 3971–3984. 11537735 10.1016/0016-7037(93)90347-y

[ece311162-bib-0018] Carraro, L. , Hartikainen, H. , Jokela, J. , Bertuzzo, E. , & Rinaldo, A. (2018). Estimating species distribution and abundance in river networks using environmental DNA. Proceedings of the National Academy of Sciences of the United States of America, 115, 11724–11729. 30373831 10.1073/pnas.1813843115 PMC6243290

[ece311162-bib-0019] Casamatta, D. A. , Johansen, J. R. , Vis, M. L. , & Broadwater, S. T. (2005). Molecular and morphological characterization of ten polar and near‐polar strains within the O*scillatoriale*s (Cyanobacteria). Journal of Phycology, 41, 421–438.

[ece311162-bib-0020] Chaudhary, A. , Haack, S. K. , Duris, J. W. , & Marsh, T. (2009). Bacterial and Archaeal phylogenetic diversity of a cold sulfur‐rich spring on the shoreline of Lake Erie, Michigan. Applied and Environmental Microbiology, 75, 5025‐36. 19542341 10.1128/AEM.00112-09 PMC2725501

[ece311162-bib-0021] Collins, R. A. , Wangensteen, O. S. , O'Gorman, E. J. , Mariani, S. , Sims, D. W. , & Genner, M. J. (2018). Persistence of environmental DNA in marine systems. Communications Biology, 1, 185. 30417122 10.1038/s42003-018-0192-6 PMC6218555

[ece311162-bib-0023] Corinaldesi, C. , Danovaro, R. , & Dell'Anno, A. (2005). Simultaneous recovery of extracellular and intracellular DNA suitable for molecular studies from marine sediments. Applied and Environmental Microbiology, 71, 46–50. 15640168 10.1128/AEM.71.1.46-50.2005 PMC544275

[ece311162-bib-0025] de Mendiburu, F. (2021). *agricolae: Statistical Procedures for Agricultural Research*. R package version 1.3‐5.

[ece311162-bib-0027] Dick, G. J. , Grim, S. L. , & Klatt, J. M. (2018). Controls on O_2_ production in cyanobacterial mats and implications for Earth's oxygenation. Annual Review of Earth and Planetary Sciences, 46, 123–147.

[ece311162-bib-0028] Domaizon, I. , Winegardner, A. , Capo, E. , Gauthier, J. , & Gregory‐Eaves, I. (2017). DNA‐based methods in paleolimnology: New opportunities for investigating long‐term dynamics of lacustrine biodiversity. Journal of Paleolimnology, 58, 1–21.

[ece311162-bib-0029] Dvořák, P. , Hašler, P. , Casamatta, D. A. , & Poulíčková, A. (2021). Underestimated cyanobacterial diversity: Trends and perspectives of research in tropical environments. Fottea, 21, 110–127.

[ece311162-bib-0030] Dvořák, P. , Jahodářová, E. , Casamatta, D. A. , Hašler, P. , & Poulíčková, A. (2018). Difference without distinction? Gaps in cyanobacterial systematics; when more is just too much. Fottea, 18, 130–136.

[ece311162-bib-0031] Eloe‐Fadrosh, E. A. , Ivanova, N. N. , Woyke, T. , & Kyrpides, N. C. (2016). Metagenomics uncovers gaps in amplicon‐based detection of microbial diversity. Nature Microbiology, 1, 1–4. 10.1038/nmicrobiol.2015.32 27572438

[ece311162-bib-0032] Esenkulova, S. , Sutherland, B. J. G. , Tabata, A. , Haigh, N. , Pearce, C. M. , & Miller, K. M. (2020). Comparing metabarcoding and morphological approaches to identify phytoplankton taxa associated with harmful algal blooms. FACETS, 5, 784–811.

[ece311162-bib-0127] Fadeev, E. , Cardozo‐Mino, M. G. , Rapp, J. Z. , Bienhold, C. , Salter, I. , Salman‐Carvalho, V. , Molari, M. , Tegetmeyer, H. E. , Buttigieg, P. L. , & Boetius, A. (2021). Comparison of two 16S rRNA primers (V3–V4 and V4–V5) for studies of Arctic microbial communities. Frontiers in Microbiology, 12, 1–11. 10.3389/fmicb.2021.637526 PMC7920977 33664723

[ece311162-bib-0033] Fawley, M. , Fawley, K. , & Cahoon, A. (2021). Finding needles in a haystack – Extensive diversity in the eustigmatophyceae revealed by community metabarcode analysis targeting the *rbc*L gene using lineage‐directed primers. Journal of Phycology, 57, 1636–1647. 34218435 10.1111/jpy.13196 PMC8530920

[ece311162-bib-0034] Filker, S. , Sommaruga, R. , Vila, I. , & Stoeck, T. (2016). Microbial eukaryote plankton communities of high‐mountain lakes from three continents exhibit strong biogeographic patterns. Molecular Ecology, 25, 2286–2301. 27029537 10.1111/mec.13633 PMC4976798

[ece311162-bib-0035] Finlay, B. J. , & Fenchel, T. (2004). Cosmopolitan metapopulations of free‐living microbial eukaryotes. Protist, 155, 237–244. 15305798 10.1078/143446104774199619

[ece311162-bib-0036] Franks, J. , & Stolz, J. F. (2009). Flat laminated microbial mat communities. Earth‐Science Reviews, 96, 163–172.

[ece311162-bib-0037] Gaylarde, C. , Gaylarde, P. M. , Copp, J. , & Neilan, B. (2004). Polyphasic detection of cyanobacteria in terrestrial biofilms. Biofouling, 20, 71–79. 15203960 10.1080/08927010410001681237

[ece311162-bib-0038] Gomez, F. J. , Mlewski, C. , Boidi, F. J. , Farías, M. E. , & Gérard, E. (2018). Calcium carbonate precipitation in diatom‐rich microbial mats: The Laguna Negra hypersaline lake, Catamarca, Argentina. Journal of Sedimentary Research, 88, 727–742.

[ece311162-bib-0040] Grim, S. L. , Stuart, D. G. , Aron, P. , Levin, N. E. , Kinsman‐Costello, L. E. , Waldbauer, J. E. , & Dick, G. J. (2023). Seasonal shifts in community composition and proteome expression in a sulfur‐cycling cyanobacterial mat. *bioRxiv* (preprint). 10.1111/1462-2920.16480 37596970

[ece311162-bib-0041] Grim, S. L. , Voorhies, A. A. , Biddanda, B. A. , Jain, S. , Nold, S. C. , Green, R. , & Dick, G. J. (2021). Omics‐inferred partitioning and expression of diverse biogeochemical functions in a low‐O_2_ cyanobacterial mat community. mSystems, 6, e0104221. 34874776 10.1128/mSystems.01042-21 PMC8651085

[ece311162-bib-0042] Guillard, R. R. L. , & Lorenzen, C. J. (1972). Yellow‐green algae with chlorophyllide C1,2. Journal of Phycology, 8, 10–14.

[ece311162-bib-0043] Haack, S. , Neff, B. , Rosenberry, D. , Savino, J. , & Lundstrom, S. (2005). An evaluation of effects of groundwater exchange on nearshore habitats and water quality of western Lake Erie. Journal of Great Lakes Research, 31, 45–63.

[ece311162-bib-0044] Hamsher, S. E. , Evans, K. M. , Mann, D. G. , Poulíčková, A. , & Saunders, G. W. (2011). Barcoding diatoms: Exploring alternatives to COI‐5P. Protist, 162, 405–422. 21239228 10.1016/j.protis.2010.09.005

[ece311162-bib-0045] Hamsher, S. E. , LeGresley, M. M. , Martin, J. L. , & Saunders, G. W. (2013). A comparison of morphological and molecular‐based surveys to estimate the species richness of *Chaetoceros* and *Thalassiosira* (Bacillariophyta), in the Bay of Fundy. PLoS One, 8, e73521. 24130665 10.1371/journal.pone.0073521 PMC3794052

[ece311162-bib-0046] Hamsher, S. E. , Verb, R. G. , & Vis, M. L. (2004). Analysis of acid mine drainage impacted streams using a periphyton index. Journal of Freshwater Ecology, 19, 13–324.

[ece311162-bib-0047] Hijmans, R. (2022). *geosphere: Spherical Trigonometry*. R package version 1.5‐18.

[ece311162-bib-0128] Illumina . (2013). 16S metagenomic sequencing library preparation. https://support.illumina.com/downloads/16s_metagenomic_sequencing_library_preparation.html

[ece311162-bib-0048] Jones, H. M. , Simpson, G. E. , Stickle, A. J. , & Mann, D. G. (2005). Life history and systematics of *Petroneis* (Bacillariophyta), with special reference to British waters. European Journal of Phycology, 40, 61–87.

[ece311162-bib-0049] Kahlert, M. , Kelly, M. , Albert, R. L. , Almeida, S. F. P. , Bešta, T. , Blanco, S. , Coste, M. , Denys, L. , Ector, L. , Fránková, M. , Hlúbiková, D. , Ivanov, P. , Kennedy, B. , Marvan, P. , Mertens, A. , Miettinen, J. , Picinska‐Fałtynowicz, J. , Rosebery, J. , Tornés, E. , … Vogel, A. (2012). Identification versus counting protocols as sources of uncertainty in diatom‐based ecological status assessments. Hydrobiologia, 695, 109–124.

[ece311162-bib-0050] Kassambara, A. (2023). *ggpubr: “ggplot2” Based Publication Ready Plots*. R package version 0.6.0.

[ece311162-bib-0051] Kermarrec, L. , Franc, A. , Rimet, F. , Chaumeil, P. , Humbert, J. F. , & Bouchez, A. (2013). Next‐generation sequencing to inventory taxonomic diversity in eukaryotic communities: A test for freshwater diatoms. Molecular Ecology Resources, 13, 607–619. 23590277 10.1111/1755-0998.12105

[ece311162-bib-0052] Kinsman‐Costello, L. E. , Sheik, C. S. , Sheldon, N. D. , Allen Burton, G. , Costello, D. M. , Marcus, D. , Uyl, P. A. D. , & Dick, G. J. (2017). Groundwater shapes sediment biogeochemistry and microbial diversity in a submerged Great Lake sinkhole. Geobiology, 15, 225–239. 27671809 10.1111/gbi.12215

[ece311162-bib-0054] Kociolek, J. P. , Kopalová, K. , Hamsher, S. E. , Kohler, T. J. , Van de Vijver, B. , Convey, P. , & McKnight, D. M. (2017). Freshwater diatom biogeography and the genus *Luticola*: An extreme case of endemism in Antarctica. Polar Biology, 40, 1185–1196.

[ece311162-bib-0055] Komárek, J. , & Anagnostidis, K. (2005). “Cyanoprokaryota; Oscillatoriales” in in Süßwasserflora von Mitteleuropa, Book 19/2. Elsevier/Spektrum.

[ece311162-bib-0056] Könneke, M. , Bernhard, A. E. , de la Torre, J. R. , Walker, C. B. , Waterbury, J. B. , & Stahl, D. A. (2005). Isolation of an autotrophic ammonia‐oxidizing marine archaeon. Nature, 437, 543–546. 16177789 10.1038/nature03911

[ece311162-bib-0058] Krammer, K. & H. Lange‐Bertalot , 1986. Bacillariophyceae. 1. Teil: Naviculaceae. VEB Gustav Fisher Verlag.

[ece311162-bib-0059] Krammer, K. & H. Lange‐Bertalot , 1988. Bacillariophyceae. 2. Teil: Epithemiaceae, Bacillariaceae, Surirellaceae. VEB Gustav Fisher Verlag.

[ece311162-bib-0060] Krammer, K. & H. Lange‐Bertalot , 1991a. Bacillariophyceae. 3. Teil: Centrales, Fragilariaceae, Eunotiaceae, Achnanthaceae. VEB Gustav Fisher Verlag.

[ece311162-bib-0061] Krammer, K. & H. Lange‐Bertalot , 1991b. Bacillariophyceae. 4. Teil: Achnanthaceae, Kritische Erganzungenzu *Navicula* (Lkleolatae) und *Gomphonema* . VEB Gustav Fisher Verlag.

[ece311162-bib-0063] Lance, R. , Klymus, K. , Richter, C. , Guan, X. , Farrington, H. , Carr, M. , Thompson, N. , Chapman, D. , & Baerwaldt, K. (2017). Experimental observations on the decay of environmental DNA from bighead and silver carps. Management of Biological Invasions, 8, 343–359.

[ece311162-bib-0064] Lear, G. , Washington, V. , Neale, M. , Case, B. , Buckley, H. , & Lewis, G. (2013). The biogeography of stream bacteria. Global Ecology and Biogeography, 22, 544–554.

[ece311162-bib-0065] Lefler, F. , Berthold, D. , & Laughinghouse, D. (2023). CyanoSeq: A database of cyanobacterial 16S rRNA sequences with curated taxonomy. Journal of Phycology, 59, 470–480. 37026389 10.1111/jpy.13335

[ece311162-bib-0066] Levi, P. S. , Starnawski, P. , Poulsen, B. , Baattrup‐Pedersen, A. , Schramm, A. , & Riis, T. (2017). Microbial community diversity and composition varies with habitat characteristics and biofilm function in macrophyte‐rich streams. Oikos, 126, 398–409.

[ece311162-bib-0067] Li, X. , Huo, S. , Zhang, J. , Ma, C. , Xiao, Z. , Zhang, H. , Xi, B. , & Xia, X. (2019). Metabarcoding reveals a more complex cyanobacterial community than morphological identification. Ecological Indicators, 107, 105653.

[ece311162-bib-0129] Lowe, R. L. (1974). Environmental requirements and pollution tolerance of freshwater diatoms. US Environmental Protection Agency, EPA‐670/4‐74‐005.

[ece311162-bib-0068] Lu, Q. , Zhang, S. , Du, J. , Liu, Q. , Dong, C. , Zhao, J. , Wang, Y. , & Yao, M. (2023). Multi‐group biodiversity distributions and drivers of metacommunity organization along a glacial–fluvial–limnic pathway on the Tibetan plateau. Environmental Research, 220, 115236. 36621545 10.1016/j.envres.2023.115236

[ece311162-bib-0069] Lundstrom, S. C. , Haack, S. K. , Neff, B. P. , Reeves, H. W. , & Rukstales, L. R. (2004). Geologic framework of two contrasting nearshore areas of Michigan, and new hypotheses for relationships among geology, ground‐water flow, water quality, and ecology. Three‐Dimensional Geologic Mapping for Groundwater Applications, 48.

[ece311162-bib-0070] Malik, H. I. , & Saros, J. E. (2016). Effects of temperature, light and nutrients on five *Cyclotella sensu lato* taxa assessed with in situ experiments in arctic lakes. Journal of Plankton Research, 38, 431–442.

[ece311162-bib-0071] Marcelino, V. R. , & Verbruggen, H. (2016). Multi‐marker metabarcoding of coral skeletons reveals a rich microbiome and diverse evolutionary origins of endolithic algae. Scientific Reports, 6, 1–9. 27545322 10.1038/srep31508 PMC4992875

[ece311162-bib-0072] Martin, M. (2011). Cutadapt removes adapter sequences from high‐throughput sequencing reads. EMBnet Journal, 17, 10–12.

[ece311162-bib-0073] Martinez Arbizu, P. (2020). pairwiseAdonis: Pairwise multilevel comparison using Adonis. R package version 0.4.1.

[ece311162-bib-0074] McGovern, C. A. , Norwich, A. R. , Thomas, A. L. , Hamsher, S. E. , Biddanda, B. A. , Weinke, A. D. , & Casamatta, D. A. (2023). Unbiased analyses of ITS folding motifs in a taxonomically confusing lineage: *Anagnostidinema visiae* sp. nov. (cyanobacteria). Journal of Phycology, 59, 619–634. 37073408 10.1111/jpy.13337

[ece311162-bib-0075] McMurdie, P. J. , & Holmes, S. (2013). Phyloseq: An R package for reproducible interactive analysis and graphics of microbiome census data. PLoS One, 8, 612–617. 10.1371/journal.pone.0061217 PMC3632530 23630581

[ece311162-bib-0076] Merz, E. , Dick, G. J. , de Beer, D. , Grim, S. , Hübener, T. , Littmann, S. , Olsen, K. , Stuart, D. , Lavik, G. , Marchant, H. K. , & Klatt, J. M. (2021). Nitrate respiration and diel migration patterns of diatoms are linked in sediments underneath a microbial mat. Environmental Microbiology, 23, 1422–1435. 33264477 10.1111/1462-2920.15345

[ece311162-bib-0077] Neilan, B. A. , Jacobs, D. , Del Dot, T. , Blackall, L. L. , Hawkins, P. R. , Cox, P. T. , & Goodman, A. E. (1997). rRNA sequences and evolutionary relationships among toxic and non‐toxic cyanobacteria of the genus *Microcystis* . International Journal of Systematic Bacteriology, 47, 693–697. 9226902 10.1099/00207713-47-3-693

[ece311162-bib-0078] Nold, S. C. , Pangborn, J. B. , Zajack, H. A. , Kendall, S. T. , Rediske, R. R. , & Biddanda, B. A. (2010). Benthic bacterial diversity in submerged sinkhole ecosystems. Applied and Environmental Microbiology, 76, 347–351. 19880643 10.1128/AEM.01186-09 PMC2798655

[ece311162-bib-0079] Nold, S. C. , Zajack, H. A. , & Biddanda, B. A. (2010). Eukaryal and archaeal diversity in a submerged sinkhole ecosystem influenced by sulfur‐rich, hypoxic groundwater. Journal of Great Lakes Research, 36, 366–375.

[ece311162-bib-0080] O'Dell, J. W. (1996). Determination of phosphorus by semi‐automated Colorimetry. In Methods for the determination of metals in environmental samples (pp. 479–495). Elsevier.

[ece311162-bib-0081] Ogle, D. H. , Doll, J. C. , Wheeler, A. P. , & Dinno, A. (2023). *FSA: Simple fisheries stock assessment methods*. R package version.

[ece311162-bib-0082] Okabe, S. , & Shimazu, Y. (2007). Persistence of host‐specific Bacteroides–Prevotella 16S rRNA genetic markers in environmental waters: Effects of temperature and salinity. Applied Microbiology and Biotechnology, 76, 935–944. 17598108 10.1007/s00253-007-1048-z

[ece311162-bib-0083] Oksanen, J. , Simpson, G. , Blanchet, F. , Kindt, R. , Legendre, P. , Minchin, P. , O'Hara, R. , Solymos, P. , Stevens, M. , Szoecs, E. , Wagner, H. , Barbour, M. , Bedward, M. , Bolker, B. , Borcard, D. , Carvalho, G. , Chirico, M. , De Caceres, M. , Durand, S. , … Weedon, J. (2022). vegan: Community ecology package. R package version 2.6‐4.

[ece311162-bib-0084] Pérez‐Burillo, J. , Valoti, G. , Witkowski, A. , Prado, P. , Mann, D. G. , & Trobajo, R. (2022). Assessment of marine benthic diatom communities: Insights from a combined morphological–metabarcoding approach in Mediterranean shallow coastal waters. Marine Pollution Bulletin, 174, 113–183. 10.1016/j.marpolbul.2021.113183 35090287

[ece311162-bib-0085] Perillo, V. L. , La Colla, N. S. , Pan, J. , Serra, A. V. , Botté, S. E. , & Cuadrado, D. G. (2022). Epibenthic microbial mats behavior as phosphorus sinks or sources in relation to biological and physicochemical conditions. Journal of Environmental Management, 314, 115079. 35447453 10.1016/j.jenvman.2022.115079

[ece311162-bib-0086] Pfaff, J. (1993). Method 300.0 determination of inorganic anions in water by ion chromatography. USEPA: Inorganic Chemistry Branch, Chemistry Research.

[ece311162-bib-0087] Pinckney, J. L. , Paerl, H. W. , & Fitzpatrick, M. (1995). Impacts of seasonality and nutrients on microbial mat community structure and function. Marine Ecology Progress Series, 123, 207–216.

[ece311162-bib-0088] Pitz, K. J. , Guo, J. , Johnson, S. B. , Campbell, T. L. , Zhang, H. , Vrijenhoek, R. C. , Chavez, F. P. , & Geller, J. (2020). Zooplankton biogeographic boundaries in the California Current System as determined from metabarcoding. PLoS One, 15, e0235159. 32584911 10.1371/journal.pone.0235159 PMC7316296

[ece311162-bib-0089] Ponader, K. C. , & Potapova, M. G. (2007). Diatoms from the genus *Achnanthidium* in flowing waters of the Appalachian Mountains (North America): Ecology, distribution and taxonomic notes. Limnologica, 37, 227–241.

[ece311162-bib-0090] Power, J. F. , Carere, C. R. , Lee, C. K. , Wakerley, G. L. J. , Evans, D. W. , Button, M. , White, D. , Climo, M. D. , Hinze, A. M. , Morgan, X. C. , McDonald, I. R. , Cary, S. C. , & Stott, M. B. (2018). Microbial biogeography of 925 geothermal springs in New Zealand. Nature Communications, 9, 2876. 10.1038/s41467-018-05020-y PMC6056493 30038374

[ece311162-bib-0091] Prieto‐Barajas, C. M. , Valencia‐Cantero, E. , & Santoyo, G. (2018). Microbial mat ecosystems: Structure types, functional diversity, and biotechnological application. Electronic Journal of Biotechnology, 31, 48–56.

[ece311162-bib-0092] Quast, C. , Pruesse, E. , Yilmaz, P. , Gerken, J. , Schweer, T. , Yarza, P. , Peplies, J. , & Glöckner, F. O. (2013). The SILVA ribosomal RNA gene database project: Improved data processing and web‐based tools. Nucleic Acids Research, 41, 590–596. 10.1093/nar/gks1219 PMC3531112 23193283

[ece311162-bib-0126] R Core Team . (2022). R: A language and environment for statistical computing. R Foundation for Statistical Computing.

[ece311162-bib-0130] Reavie, E. D. , Axler, R. P. , Sgro, G. V. , Danz, N. P. , Kingston, J. C. , Kireta, A. R. , Brown, T. N. , Hollenhorst, T. P. , & Ferguson, M. J. (2006). Diatom‐based weighted‐averaging transfer functions for Great Lakes coastal water quality: Relationships to watershed characteristics. Journal of Great Lakes Research, 32, 321–347.

[ece311162-bib-0093] Remane, A. , & Schlieper, C. (1971). Biology of brackish water. E. Schweizerbart'sche Verlagsbuchhandlung.

[ece311162-bib-0094] Ribeiro, K. F. , Duarte, L. , & Crossetti, L. O. (2018). Everything is not everywhere: A tale on the biogeography of cyanobacteria. Hydrobiologia, 820, 23–48.

[ece311162-bib-0095] Richlen, M. L. , & Barber, P. H. (2005). A technique for the rapid extraction of microalgal DNA from single live and preserved cells. Molecular Ecology Notes, 5, 688–691.

[ece311162-bib-0096] Rimet, F. , & Bouchez, A. (2012). Biomonitoring river diatoms: Implications of taxonomic resolution. Ecological Indicators, 15, 92–99.

[ece311162-bib-0097] Rimet, F. , Gusev, E. , Kahlert, M. , Kelly, M. G. , Kulikovskiy, M. , Maltsev, Y. , Mann, D. G. , Pfannkuchen, M. , Trobajo, R. , Vasselon, V. , Zimmermann, J. , & Bouchez, A. (2019). Diat.Barcode, an open‐access curated barcode library for diatoms. Scientific Reports, 9, 15116. 31641158 10.1038/s41598-019-51500-6 PMC6805954

[ece311162-bib-0098] Rimet, F. , Trobajo, R. , Mann, D. G. , Kermarrec, L. , Franc, A. , Domaizon, I. , & Bouchez, A. (2014). When is sampling complete? The effects of geographical range and marker choice on perceived diversity in *Nitzschia palea* (Bacillariophyta). Protist, 165, 245–259. 24739436 10.1016/j.protis.2014.03.005

[ece311162-bib-0099] Rippka, R. (1988). [1] Isolation and purification of cyanobacteria. In Methods in enzymology (Vol. 167, pp. 3–27). Academic Press. 3148836 10.1016/0076-6879(88)67004-2

[ece311162-bib-0100] Ruberg, S. , Kendall, S. , Biddanda, B. , Black, T. , Nold, S. , Lusardi, W. , Green, R. , Casserley, T. , Smith, E. , Sanders, G. , Lang, G. , & Constant, S. (2008). Observations of the Middle Island Sinkhole in Lake Huron—A unique hydrogeologic and glacial creation of 400 million years. Marine Technology Society Journal, 42, 12–21.

[ece311162-bib-0101] Saros, J. E. , & Anderson, N. J. (2015). The ecology of the planktonic diatom *Cyclotella* and its implications for global environmental change studies. Biological Reviews, 90, 522–541. 24917134 10.1111/brv.12120

[ece311162-bib-0102] Shogren, A. J. , Tank, J. L. , Egan, S. P. , August, O. , Rosi, E. J. , Hanrahan, B. R. , Renshaw, M. A. , Gantz, C. A. , & Bolster, D. (2018). Water flow and biofilm cover influence environmental DNA detection in recirculating streams. Environmental Science & Technology, 52, 8530–8537. 29995389 10.1021/acs.est.8b01822

[ece311162-bib-0103] Singer, G. , Besemer, K. , Schmitt‐Kopplin, P. , Hödl, I. , & Battin, T. J. (2010). Physical heterogeneity increases biofilm resource use and its molecular diversity in stream mesocosms. PLoS One, 5, e9988. 20376323 10.1371/journal.pone.0009988 PMC2848676

[ece311162-bib-0104] Snider, M. J. , Biddanda, B. A. , Lindback, M. , Grim, S. L. , & Dick, G. J. (2017). Versatile photophysiology of compositionally similar cyanobacterial mat communities inhabiting submerged sinkholes of Lake Huron. Aquatic Microbial Ecology, 79, 63–78.

[ece311162-bib-0105] Stal, L. J. (1995). Physiological ecology of cyanobacteria in microbial mats and other communities. The New Phytologist, 131, 1–32. 33863161 10.1111/j.1469-8137.1995.tb03051.x

[ece311162-bib-0106] Steckbauer, A. , Duarte, C. M. , Carstensen, J. , Vaquer‐Sunyer, R. , & Conley, D. J. (2011). Ecosystem impacts of hypoxia: Thresholds of hypoxia and pathways to recovery. Environmental Research Letters, 6, 025003.

[ece311162-bib-0107] Stepanek, J. G. , Mayama, S. , & Kociolek, J. P. (2015). Description and phylogenetic position of *Amphora aliformis* (Bacillariophyta), a new species from Tokyo Bay. Phycologia, 54, 78–86.

[ece311162-bib-0108] Stevenson, R. J. , Bothwell, M. L. , Lowe, R. L. , & Thorp, J. H. (1996). Algal ecology: Freshwater benthic ecosystem. Academic Press.

[ece311162-bib-0110] Strickler, K. M. , Fremier, A. K. , & Goldberg, C. S. (2015). Quantifying effects of UV‐B, temperature, and pH on eDNA degradation in aquatic microcosms. Biological Conservation, 183, 85–92.

[ece311162-bib-0111] Šupraha, L. , Klemm, K. , Gran‐Stadniczeñko, S. , Hörstmann, C. , Vaulot, D. , Edvardsen, B. , & Uwe, J. (2022). Diversity and biogeography of planktonic diatoms in Svalbard fjords: The role of dispersal and Arctic endemism in phytoplankton community structuring. Elementa: Science of the Anthropocene, 10, 1.

[ece311162-bib-0112] Tsuji, S. , Ushio, M. , Sakurai, S. , Minamoto, T. , & Yamanaka, H. (2017). Water temperature‐dependent degradation of environmental DNA and its relation to bacterial abundance. PLoS One, 12, e0176608. 28448613 10.1371/journal.pone.0176608 PMC5407774

[ece311162-bib-0113] USEPA Method 370.1 . (1978). *Methods for the chemical analysis of water and wastes (MCAWW) EPA/600/4‐79/020*. EPA Method, 375.

[ece311162-bib-0114] Van de Vijver, B. , Williams, D. M. , Schuster, T. M. , Kusber, W. H. , Cantonati, M. , Wetzel, C. E. , & Ector, L. (2022). Analysis of the *Fragilaria rumpens* complex (Fragilariaceae, Bacillariophyta) with the description of two new species. Fottea, 22, 93–121.

[ece311162-bib-0115] Varliero, G. , Lebre, P. H. , Stevens, M. I. , Czechowski, P. , Makhalanyane, T. , & Cowan, D. A. (2023). The use of different 16S rRNA gene variable regions in biogeographical studies. Environmental Microbiology Reports, 15, 216–228. 36810880 10.1111/1758-2229.13145 PMC10464692

[ece311162-bib-0116] Vasselon, V. , Bouchez, A. , Rimet, F. , Jacquet, S. , Trobajo, R. , Corniquel, M. , Tapolczai, K. , & Domaizon, I. (2018). Avoiding quantification bias in metabarcoding: Application of a cell biovolume correction factor in diatom molecular biomonitoring. Methods in Ecology and Evolution, 9, 1060–1069.

[ece311162-bib-0117] Vasselon, V. , Rimet, F. , Tapolczai, K. , & Bouchez, A. (2017). Assessing ecological status with diatoms DNA metabarcoding: Scaling‐up on a WFD monitoring network (Mayotte Island, France). Ecological Indicators, 82, 1–12.

[ece311162-bib-0118] von Wintzingerode, F. , Göbel, U. B. , & Stackebrandt, E. (1997). Determination of microbial diversity in environmental samples: Pitfalls of PCR‐based rRNA analysis. FEMS Microbiology Reviews, 21, 213–229. 9451814 10.1111/j.1574-6976.1997.tb00351.x

[ece311162-bib-0119] Voorhies, A. A. , Biddanda, B. A. , Kendall, S. T. , Jain, S. , Marcus, D. N. , Nold, S. C. , Sheldon, N. D. , & Dick, G. J. (2012). Cyanobacterial life at low O_2_: Community genomics and function reveal metabolic versatility and extremely low diversity in a Great Lakes sinkhole mat. Geobiology, 10, 250–267. 22404795 10.1111/j.1472-4669.2012.00322.x

[ece311162-bib-0120] Walters, W. , Hyde, E. R. , Berg‐Lyons, D. , Ackermann, G. , Humphrey, G. , Parada, A. , Gilbert, J. A. , Jansson, J. K. , Caporaso, J. G. , Fuhrman, J. A. , Apprill, A. , & Knight, R. (2015). Improved bacterial 16S rRNA gene (V4 and V4‐5) and fungal internal transcribed spacer marker gene primers for microbial community surveys. mSystems, 1, e00009‐15. 27822518 10.1128/mSystems.00009-15 PMC5069754

[ece311162-bib-0121] Wehr, J. D. , Sheath, R. G. , & Kociolek, J. P. (2015). Freshwater algae of North America: Ecology and classification. Elsevier.

[ece311162-bib-0122] Weltz, K. , Lyle, J. M. , Ovenden, J. , Morgan, J. A. T. , Moreno, D. A. , & Semmens, J. M. (2017). Application of environmental DNA to detect an endangered marine skate species in the wild. PLoS One, 12, e0178124. 28591215 10.1371/journal.pone.0178124 PMC5462358

[ece311162-bib-0123] Wolf, D. I. , & Vis, M. L. (2019). Stream algal biofilm community diversity along an acid mine drainage recovery gradient using multimarker metabarcoding. Journal of Phycology, 56, 11–22. 31621078 10.1111/jpy.12935

[ece311162-bib-0124] Zimmermann, J. , Glöckner, G. , Jahn, R. , Enke, N. , & Gemeinholzer, B. (2015). Metabarcoding vs. morphological identification to assess diatom diversity in environmental studies. Molecular Ecology Resources, 15, 526–542. 25270047 10.1111/1755-0998.12336

